# CAGE Binds to Beclin1, Regulates Autophagic Flux and CAGE-Derived Peptide Confers Sensitivity to Anti-cancer Drugs in Non-small Cell Lung Cancer Cells

**DOI:** 10.3389/fonc.2018.00599

**Published:** 2018-12-10

**Authors:** Minjeong Yeon, Jaewhan Byun, Hyuna Kim, Misun Kim, Hyun Suk Jung, Doyong Jeon, Youngmi Kim, Dooil Jeoung

**Affiliations:** ^1^Department of Biochemistry, Kangwon National University, Chunchon, South Korea; ^2^L-Base Company, Seoul, South Korea; ^3^Institute of New Frontier Research, College of Medicine, Hallym University, Chunchon, South Korea

**Keywords:** anti-cancer drug-resistance, autophagy, cancer/testis antigen CAGE, CAGE-derived peptide, non-small cell lung cancers

## Abstract

The objective of this study was to determine the role of CAGE, a cancer/testis antigen, in resistance of non-small cell lung cancers to anti-cancer drugs. Erlotinib-resistant PC-9 cells (PC-9/ER) with EGFR mutations (ex 19 del + T790M of EGFR), showed higher level of autophagic flux than parental sensitive PC-9 cells. Erlotinib and osimertinib increased autophagic flux and induced the binding of CAGE to Beclin1 in PC-9 cells. The inhibition or induction of autophagy regulated the binding of CAGE to Beclin1 and the responses to anti-cancer drugs. CAGE showed binding to HER2 while HER2 was necessary for binding of CAGE to Beclin1. CAGE was responsible for high level of autophagic flux and resistance to anti-cancer drugs in PC-9/ER cells. A peptide corresponding to the DEAD box domain of CAGE, ^266^AQTGTGKT^273^, enhanced the sensitivity of PC-9/ER cells to erlotinib and osimertinib, inhibited the binding of CAGE to Beclin1 and regulated autophagic flux in PC-9/ER cells. Mutant CAGE-derived peptide ^266^AQTGTGAT^273^ or ^266^AQTGTGKA^273^ did not affect autophagic flux or the binding of CAGE to Beclin1. AQTGTGKT peptide showed binding to CAGE, but not to Beclin1. FITC-AQTGTGKT peptide showed co-localization with CAGE. AQTGTGKT peptide decreased tumorigenic potentials of PC-9/ER and H1975 cells, non-small cell lung cancer (NSCLC) cells with EGFR mutation (L885R/T790M), by inhibiting autophagic fluxand inhibiting the binding of CAGE to Beclin1. AQTGTGKT peptide also enhanced the sensitivity of H1975 cells to anti-cancer drugs. AQTGTGKT peptide showed tumor homing potential based on *ex vivo* homing assays of xenograft of H1975 cells. AQTGTGKT peptide restored expression levels of miR-143-3p and miR-373-5p, decreased autophagic flux and conferred sensitivity to anti-cancer drugs. These results present evidence that combination of anti-cancer drug with CAGE-derived peptide could overcome resistance of non-small cell lung cancers to anti-cancer drugs.

## Introduction

CAGE, a cancer/testis antigen, shows wide expression among tumor tissues while its expression is limited to testis among normal tissues (1). CAGE is present in the sera of patients with various cancers (1, 2). It has been shown that CAGE is predominantly reacting with sera from gastric cancer patients, not healthy controls (3). The expression of CAGE is under epigenetic regulation (4). X-ray structure of CAGE reveals that CAGE belongs to RNA helicase (5).

CAGE possesses oncogenic potential and promotes cell cycle progression by inducing AP-1- and E2F-dependent expression of cyclins D1 and E (6). It stimulates angiogenesis (7, 8), binds to HDAC2 and confers resistance to anti-cancer drugs in various cancer cells (9). Histone deacetylase-3 directly regulates the expression of CAGE and pEGFR^Y845^ to confer sensitivity to anti-cancer drugs (10). MiR-200b and CAGE can form a feedback regulatory loop and regulate the response to microtubule-targeting drugs, as well as the invasion, tumorigenic potential, and angiogenic potential (7). Peptides corresponding to the DEAD box helicase domain of CAGE such as AQTGTGKT shows anti-cancer activity by preventing CAGE from binding to GSK3β (11).

CAGE directly regulates SOX-2 expression and cancer stem cell-like properties in anti-cancer drug-resistant melanoma cells (12). It promotes stem cell-like properties, autophagy, and confers resistance to anti-cancer drugs in breast cancer cells (13).

Epidermal growth factor receptor-tyrosine-kinase inhibitors (EGFR-TKIs) have been used to treat non-small cell lung cancers. Acquired resistance to EGFR-TKIs such as gefitinib and erlotinib is a critical obstacle in the treatment of EGFR mutant-positive non-small cell lung cancer (NSCLC). Acquired EGFR mutation (T790M) can lead to resistance to gefitinib treatment (14). EGFR mutation (L858R/L718V) confers resistance to osimertinib. However, it retains sensitivity to second generation TKI afatinib (14). Erlotinib-resistance is associated with EGFR kinase domain (15). EHD1, a protein of the C-terminal Eps15 homology domain-containing (EHD) family, is involved in EGFR-TKI resistance. Lower EHD1 expression improves both EGFR-TKIs sensitivity and progression-free survival in NSCLC patients (16). Decreased expression of EGFR by autophagosome enhances efficacy of EGFR-THI such as erlotinib on PC-9/ER cells with T790M EGFR mutation (17). Erlotinib resistance driven by EGFR mutation (T790M) in non-small cell lung cancer is associated with decreased glutathione level (18). PC-9/ER cells show activation of MET while MET can enhance the migration and invasion potential of PC-9/ER cells (19). The activation of bypass signals such as MET and AXL can lead to EGFR-TKI resistance (20).

In the present study, we found that CAGE could regulate autophagic flux and responses to anti-cancer drugs in non-small cell lung cancer cells with EGFR mutations such as PC-9/ER cells and H1975 cells. CAGE showed binding to Beclin1, a mediator of autophagy, in PC-9/ER cells, and H1975 cells. CAGE-derived AQTGTGKT peptide showed binding to CAGE and conferred sensitivity to anti-cancer drugs by inhibiting the interaction between CAGE and Beclin1. AQTGTGKT peptide showed homing to tumor tissue to decrease tumorigenic potentials of non-small cell lung cancer cells. We identified targets of AQTGTGKT peptide and showed effects of these miRNAs on autophagic flux and responses to anti-cancer drugs. Roles of CAGE and CAGE-derived peptide in regulating responses to anti-cancer drugs such as erlotinib and osimertinib in non-small cell lung cancer cells with EGFR mutations were revealed for the first time here.

## Materials and Methods

### Materials

An enhanced chemiluminescence (ECL) kit was purchased from Amersham Biosciences. Lipofectamine and PlusTM reagent were purchased from Invitrogen. SiRNAs used in this study were purchased from Bioneer (Daejeon, Korea). MiRNA inhibitors used in this study were purchased from Bioneer Company. Chemicals used in this study were purchased from Sigma Chemical Company. Anti-mouse and anti-rabbit IgG-horse radish peroxidase conjugate antibodies were purchased from Pierce Company (Rockford, IL). All other antibodies used in this study were purchased from Santa Cruz Company and Cell Signaling Company.

### Cell Lines and Cell Culture

Cancer cell lines used in this study were cultured in Dulbecco's modified minimal essential medium (Invitrogen) supplemented with heat-inactivated 10% fetal bovine serum (Invitrogen) and antibiotics at 37°C in a humidified incubator with a mixture of 95% air and 5% CO_2_.

### Peptides

Peptides used in this study were commercially synthesized by Peptron Company (Daejon, Koea). The FITC-conjugated AQTGTGKT, and Biotin-AQTGTGKT peptide were purified by high performance liquid chromatography and their sequence and structure were confirmed by mass spectrometry. To examine whether AQTGTGKT peptide binds to CAGE or Beclin1, biotin-AQTGTGKT peptide (Peptron, Daejeon, Korea) was transfected with Lipofectamin and PlusTM reagent (Invitrogen, San Diego, CA). After incubation for 48 h, whole-cell extracts were incubated with anti-biotin antibody (2 μg/ml) for 12 h at 4°C and immune complexes were precipitated with streptavidin-linked agarose beads for 30 min at 4°C. After five washes with lysis buffer, the bound proteins were eluted by boiling in 2X Laemli SDS loading buffer and were then subjected to SDS-PAGE followed by immunoblotting analysis with anti-CAGE antibody or anti-Beclin1 antibody.

### Cell Viability Determination

The cells were assayed for their growth activity using the 3-(4, 5-dimethylthiazol-2-yl) - 2, 5-diphenyltetrazolium bromide (Sigma). Viable cell number counting was carried out by trypan blue exclusion assays.

### Immunoblot and Immunoprecipitation

Immunoblot and immunoprecipitation were performed according to the standard procedures (12). For analysis of proteins from tumor tissues, frozen samples were ground to a fine powder using a mortar and pestle over liquid nitrogen. Proteins were solubilized in RIPA buffer containing protease inhibitors, and insoluble material was removed by centrifugation.

### Chemo Invasion and Migration Assays

The invasive potential was determined by using a transwell chamber system with 8-μm pore polycarbonate filter inserts (CoSTAR, Acton, MA). The lower and upper sides of the filter were coated with gelatin and matrigel, respectively. Trypsinized cells (5 × 10^3^) in the serum-free RPMI 1,640 medium containing 0.1% bovine serum albumin were added to each upper chamber of the transwell. RPMI 1,640 medium supplemented with 10% fetal bovine serum was placed in the lower chamber and cells were incubated at 37°C for 16 h. The cells were fixed with methanol and the invaded cells were stained and counted. Results were analyzed for statistical significance using the Student's *t*-test. Differences were considered significant when *p* < 0.05. For determination of migration potential, the lower sides of the filters were coated with gelatin. To determine the effect of AQTGTGKT peptide on the invasion potential, PC-9/ER or H1975 cells were transfected with AQTGTGKT (10 μM) for 24 h.

### Immunofluorescence Staining

Cells were seeded on 10-mm coverslips at a density of 2 × 10^5^ cells/35-mm plate. Twenty-four hours after plating, cells were washed and fixed with 4% paraformaldehyde for 15 min at room temperature, and rinsed with cold PBS (pH 7.4). After blocking with goat serum (10%) in 0.1 % BSA/ PBS, primary antibody to LC3 (Cell Signaling, 1:200) or CAGE (AbCam, 1:100) was added and cells were incubated at 4°C for 24 h. After washing with PBS, slides were incubated with anti-rabbit Alexa Fluor 488 (for LC3) or anti-mouse Alexa Fluor 488 (for CAGE) secondary antibodies for 1.5 h at RT. After removal of antibodies, cells were washed with PBS and stained with DAPI and mounted with mounting medium. Fluorescence staining was visualized using confocal microscopy.

### Transfection

Transfections were performed according to the manufacturer's instructions. Lipofectamine and Plus reagents (Invitrogen) were used. For miR-143-3p or miR-373-5p knockdown, cells were transfected with 10 nM oligonucleotide (inhibitor) with Lipofectamine 2000 (Invitrogen), according to the manufacturer's protocol. The sequences used were 5′- UGAGAUGAAGCACUGUAGCUC-3′ (miR-143-3p inhibitor), 5′-ACUCAAAAUGGGGGCGCUUUCC−3′(miR-373-5p inhibitor) and 5′-TAACACGTCTATACGCCCA−3′ (control inhibitor).

### RNA Extraction and Quantitative Real Time PCR

Total miRNA was isolated using the *mir*Vana miRNA isolation kit (Ambion). MiRNA was extended by a poly (A) tailing reaction using the A-Plus poly (A) polymerase tailing kit (Cell Script). CDNA was synthesized from miRNA with poly(A) tail using a poly (T) adaptor primer and qScriptTM reverse transcriptase (Quanta Biogenesis). Expression levels of miR-143-3p was quantified with a SYBR Green qRT-PCR kit (Ambion) using a miRNA-specific forward primer and a universal poly (T) adaptor reverse primer. The expression of miR-143-3p was defined based on the threshold (*C*_*t*_), and relative expression levels were calculated as 2^−((Ct*ofmiR-143-3p*)−(*CtofU*6))^ after normalization with reference to expression of U6 small nuclear RNA. For quantitative PCR, SYBR PCR Master Mix (Applied Biosystems) was used in a CFX96 real time system thermocycler (Bio-Rad).

### Micro RNA Array

The miRNA array kit was purchased from Koma Biotech (Seoul, Korea). MiRNA array analysis was performed according to the protocols provided by the manufacturer (Koma Biotech).

### Internalization Experiments

PC-9/ER cells (3 × 10^5^) or H1975 cells (3 × 10^5^) were seeded in 35 mm culture dishes. After 24 h of incubation, medium was replaced by fresh medium and FITC-conjugated AQTGTGKT peptide (10 μM) was added to the cells. The FITC-AQTGTGKT peptide was incubated with the tumor cells for various time intervals. The medium was then removed and the cells were washed with 1 ml PBS before being analyzed by confocal imaging on an inverted microscope with a confocal laser scanning unit. Unlabeled AQTGTGKT peptide was employed as negative control to determine auto fluorescence of tumor cells. Processed serial sections were constructed into three-dimensional images using VoxelView (Vital Images Ltd., Fairfield, IA) on a Silicon Graphics Indy workstation (Mountain View, CA).

### *In vivo* Tumorigenic Potential

Athymic nude mice (BALB/c nu/nu, 5–6-week-old females) were obtained from Orient Bio Inc. (Seoul, Korea) and were maintained in a laminar air-flow cabinet under aseptic conditions. PC-9/ER cells (1 × 10^6^) were injected subcutaneously into the dorsal flank area of the mice. Tumor volume was determined by direct measurement with calipers and calculated by the following formula: length × width × height × 0.5. To determine the effect of AQTGTGKT peptide corresponding to the DEAD box helicase domain of CAGE on the tumorigenic potential of PC-9/ER, each peptide (50 μg, 100 μg, or 200 μg/mouse) was injected via tail vein five times in a total of 41 days. Each peptide was injected when tumors reached a certain size (100~150 mm^3^). To determine the effect of AQTGTGKT peptide on the tumorigenic potential of H1975 cells, each peptide (100 μg or 200 μg/mouse) was injected via tail vein five times in a total of 31 days. All animal experiments were approved by the Institutional Animal Care and Use Committee (IACUC) of Kangwon National University (KIACUC-160329-2).

### *Ex vivo* Homing Assays

Athymic nude mice (BALB/c nu/nu, 5–6-week-old females) were obtained from Orient Bio Inc. (Seoul, Korea) and were maintained in a laminar air-flow cabinet under aseptic conditions. H1975 cells (1 × 10^6^) were injected subcutaneously into the dorsal flank area of the mice Following the establishment of sizeable tumor (200~300 mm^3^), tumor bearing mice were given intravenous injection of PBS or FITC-AQTGTGKT peptide (50 μg, 100 μg). FITC-AQTGTGKT peptide was allowed to circulate for 6 and 12 h. Tumors and control organs were excised after the injection of the fluorescent peptide and examined for fluorescence using a versatile bio-imaging system (Davinch-*in vivo* Imaging System; Davinch-K, Seoul, Korea). Images were acquired using Davinch *in vivo* imaging system with excitation at 490 nm and the emitted fluorescence was collected through a long-pass filter (520 nm). Data were analyzed by Davinch Invivo software (Davinch-K, Seoul, Korea).

### Immunohistochemical Staining

Immunohistochemical staining of lung tissues was also performed using an established avidin-biotin detection method (Vectastain ABC kit, Vector Laboratories Inc., Burlingame, CA). Briefly, 4–6 μm-thick sections of the paraffin-embedded tissue blocks were cut, mounted on positively charged glass slides, and dried in an oven at 56°C for 30 min. The sections were deparaffinized in xylene and then rehydrated in graded ethanol and water. Endogenous peroxidase was blocked by incubation in 3% (v/v) hydrogen peroxide for 15 min. Antigen retrieval was accomplished by pretreatment of the sections with citrate buffer at pH 6.0 for 20 min at 56°C in a microwave oven and then allowing the sections to cool for 30 min. Nonspecific endogenous protein binding was blocked using 1% bovine serum albumin (BSA). The sections were then incubated with primary antibodies overnight at 4°C. The following primary antibodies were used for single and double staining: anti-CAGE (1:100, AbCam), anti-pAMPK^Thr172^ (1:200, Cell Signaling), p62 (AbCam, 1:500), or anti-ATG5 (1:200, Santa Cruz Biotechnology). After washing, biotinylated secondary antibodies were applied at 1:100 or 1:200 dilutions for 1 h. The color was developed with diaminobenzidine (Vector Laboratories, Inc.). Sections were counterstained with Mayer's hematoxylin. Sections incubated without primary antibody served as controls. To visualize tissue mast cells, the sections were stained with 0.1% olivine blue (Sigma) in 0.1 N HCl for 15 min.

### Statistical Analysis

Statistical differences in this were determined by using the Student's *t*-test. *P* ≤ 0.05 was considered statistically significant.

## Results

### Autophagic Flux Is Closely Associated With Responses to Anti-cancer Drugs

**Figure 1:**
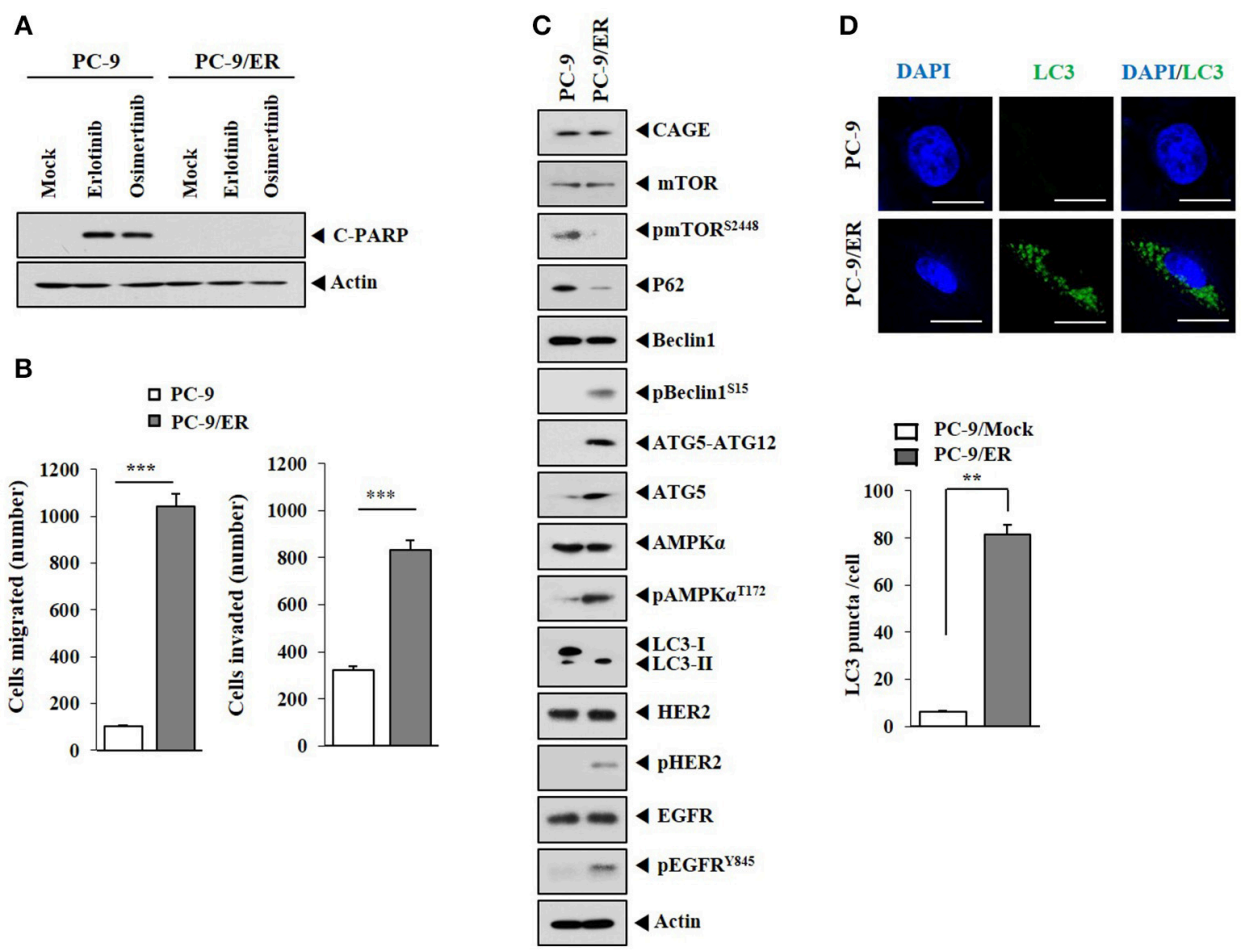
Autophagic flux is closely associated with responses to anti-cancer drugs. **(A)** Indicated cancer cells were treated without or with erlotinib (20 μM) or osimertinib (5 μM) for 24 h, followed by immunoblot. C-PARP denotes cleaved PARP. **(B)** Migration and invasion potentials of PC-9 and PC-9/ER cells were determined. ^***^p < 0.0005. **(C)** Cell lysates from the indicated cancer cells were subjected to immunoblot. **(D)** LC3 puncta expression in PC-9 and PC-9/ER cells was determined by immunofluorescence staining. ^**^p < 0.005.

Previous reports have suggested a close relationship between anti-cancer drug-resistance and autophagic flux (21–23). PC-9/ER, erlotinib-resistant non-small cell lung cancer cells with mutations in EGFR (ex 19 del + T790M of EGFR), unlike parental erlotinib-sensitive PC-9 cells, did not show cleavage of PARP in response to anti-cancer drugs such as erlotinib or osimertinib (Figure 1A). PC-9/ER cells showed higher invasion and migration potential (Figure 1B) and higher levels of autophagic flux such as pBeclin1^Ser15^, pAMPKα^Thr172^, ATG5, and LC3-II than PC-9 cells (Figure 1C). PC-9/ER cells showed higher expression level of pHER2 and pEGFR^Y845^ than PC-9 cells (Figure 1C). The expression level of CAGE in anti-cancer drug-resistant melanoma cells is known to be higher than that in anti-cancer drug-sensitive parental cells (9, 11). However, the expression level of CAGE, a cancer/testis antigen, in PC-9/ER cells did not show difference from that in PC-9 cells (Figure 1C). Inactivation of mTOR and activation of AMPK are associated with autophagy (22). Autophagy induction by inhibition of mTOR signaling can result in anti-cancer drug-resistance in head and neck squamous cell carcinoma cells (24). PC-9/ER cells showed lower expression level of pmTOR^Ser2448^ than PC-9 cells (Figure 1C). PC-9/ER cells showed higher LC3 puncta expression than PC-9 cells (Figure 1D). PC-9/ER cells showed lower expression level of p62 than PC-9 cells (Figure 1C). The decreased expression of p62, an autophagic receptor, is associated with autophagy (22). Down-regulation of p62 can increase the expression of LC3-II (25). The down-regulation of p62 in the present study increased autophagic flux, but decreased the expression of pmTOR^Ser2448^ in PC-9 cells (Figure 1D). However, down-regulation of p62 did not affect the expression of CAGE in PC-9 cells (Supplementary Figure 1). Thus, anti-cancer drug-resistance resulting from repeated exposure is closely associated with autophagic flux.

### Anti-cancer Drugs Increase Autophagic Flux

**Figure 2:**
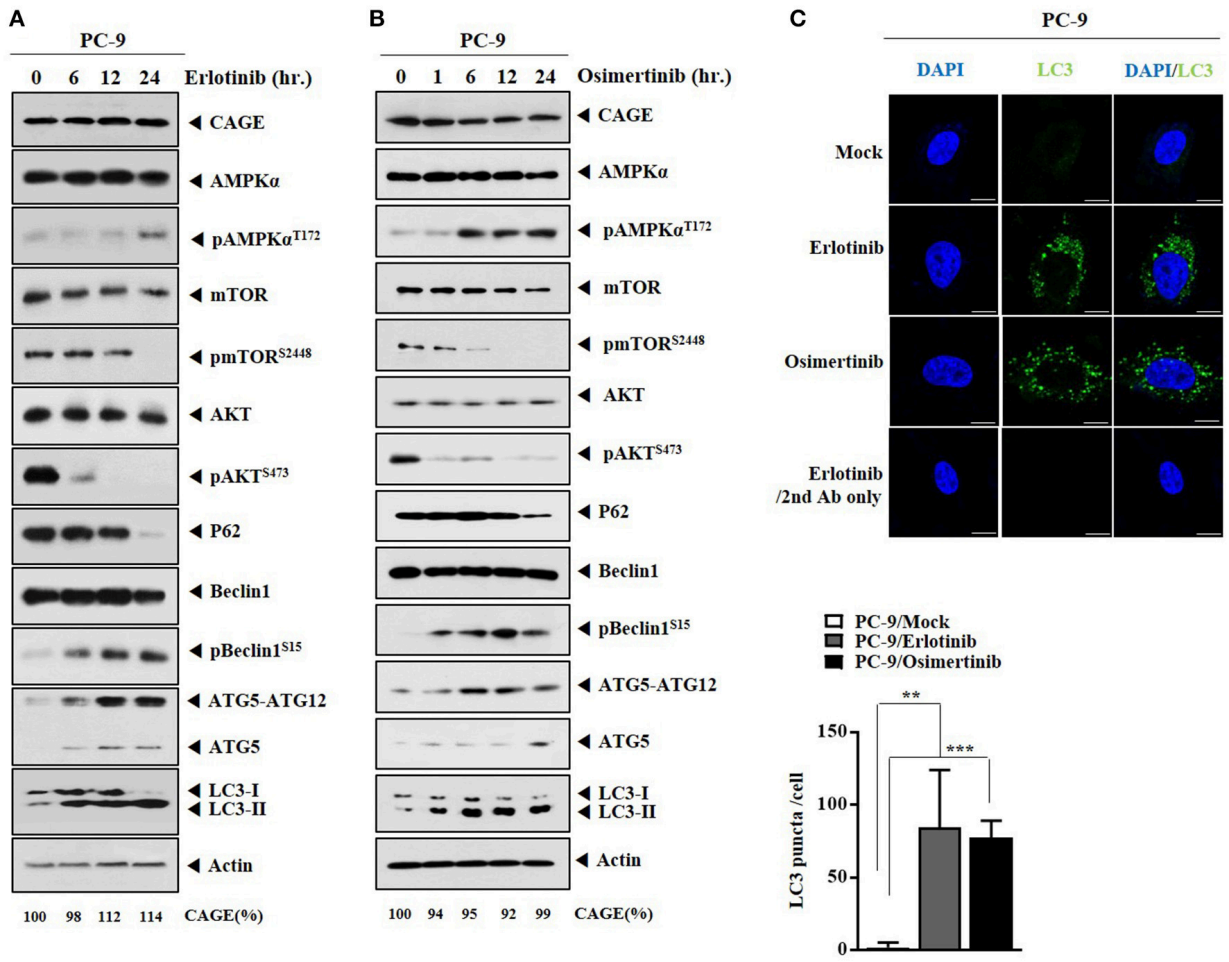
Anti-cancer drugs increase autophagic flux in PC-9 cells. **(A)** PC-9 cells were treated with erlotinib (20 μM) for various time intervals. Cells lysates prepared at each time point were subjected to immunoblot. CAGE protein level was quantified by densitometry using Image J software (Image 1.40 g, NIH, USA) and normalized to β-actin levels. **(B)** Same as **(A)** except that PC-9 cells were treated with osimertinib (5 μM) for various time intervals. **(C)** PC-9 cells were treated with erlotinib (20 μM) or osimertinib (5 μM) for 24 h. LC3 puncta expression was determined. ^**^p < 0.005; ^***^p < 0.0005. Scale bar, 10 μm.

We examined whether anti-cancer drugs could regulate autophagic flux. Erlotinib (Figure 2A) and osimertinib (Figure 2B) increased autophagic flux, but decreased expressions levels of pmTOR^Ser2448^, pAKT^Ser473^, and p62 in PC-9 cells (Figures 2A,B). Erlotinib (Figure 2A) or osimertinib (Figure 2B) did not affect the expression of CAGE in PC-9 cells. Down-regulation of EGFR protein either by siRNA or by a synthetic EGFR-down regulating peptide (Herdegradin) can kill prostate and ovarian cancer cells via selective mitophagy by activating the mTORC2/Akt axis (26). Erlotinib can decrease the expression of pAKT in PC-9 cells (19). Erlotinib and osimertinib increased LC3 puncta expression in PC-9 cells (Figure 2C). Thus, autophagic flux can be a target of anti-cancer drugs.

### Rapamycin Confers Resistance to Anti-cancer Drugs and Increases Autophagic Flux

We next examined whether rapamcyin, an inducer of autophagy, could confer resistance to anti-cancer drug-resistance in non-small cell lung cancer cells. Rapamycin increased autophagic flux in PC-9 cells, but decreased the expression of pmTOR^Ser2448^ (Supplementary Figure 2A). However, rapamycin did not affect the expression of CAGE (Supplementary Figure 2A). Rapamycin increased LC3 puncta expression in PC-9 cells (Supplementary Figure 2B). Rapamycin prevented erlotinib and osimertinib from cleaving PARP in PC-9 cells (Supplementary Figure 2C). Based on the fact that anti-cancer drugs also increased autophagic flux, it is reasonable that induction of autophagy *per se* may not stimulate anti-cancer drug-resistance in non-small cell lung cancer cells.

### Inhibition of Autophagy Targets CAGE, Induces Binding of CAGE to Beclin1 and Confers Sensitivity to Anti-cancer Drugs in PC-9/ER Cells

**Figure 3:**
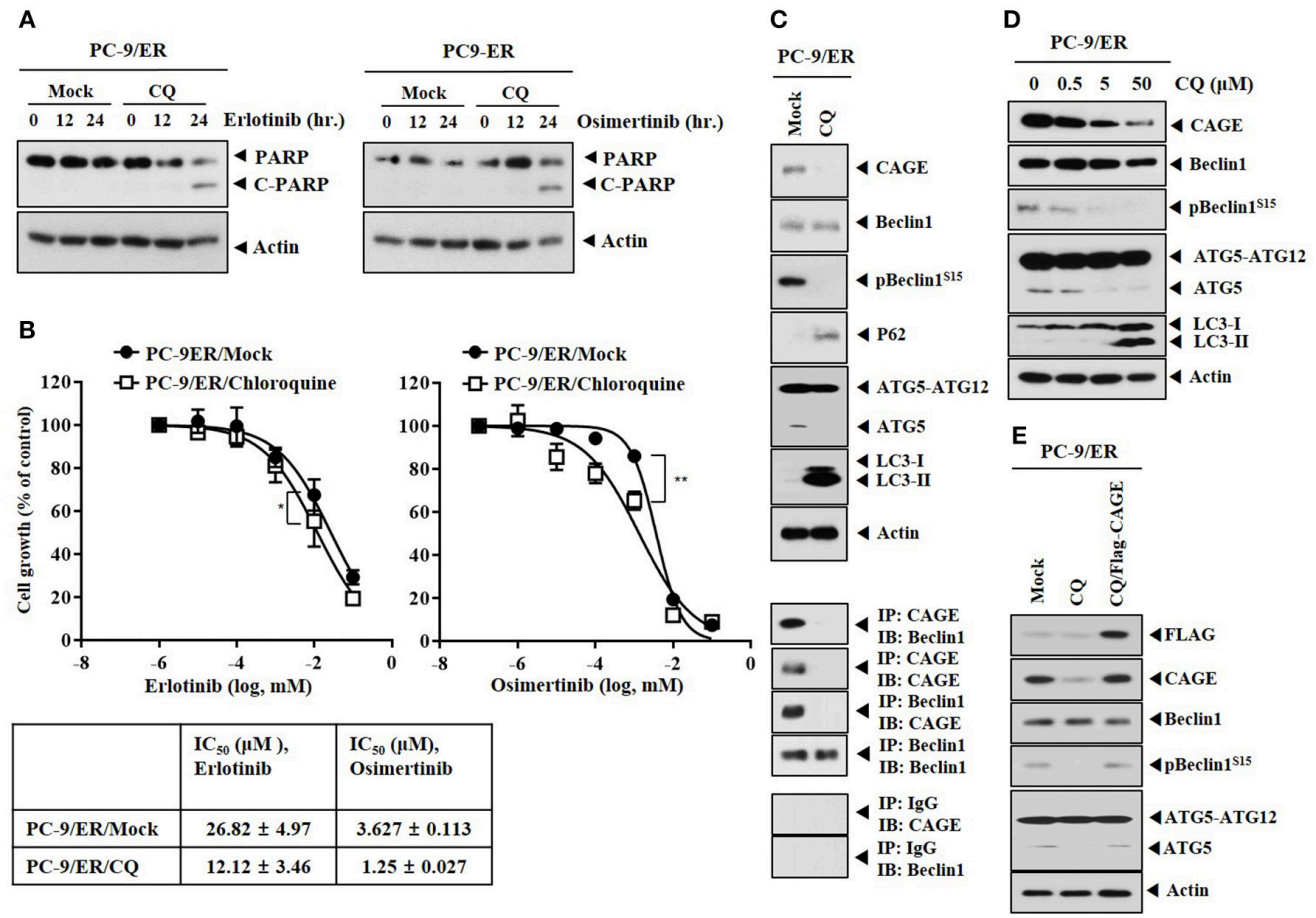
Chloroquine decreases the expression of CAGE and enhances the sensitivity to anti-cancer drugs. **(A)** PC-9/ER cells were treated without or with CQ (50 μM) for 24 h, followed by treatment with erlotinib (20 μM) or osimertinib (5 μM) for 24 h. Immunoblot was then performed. **(B)** PC-9/ER cells were treated without or with CQ (50 μM) for 24 h, followed by treatment with various concentrations of erlotinib or osimertinib for 24 h. MTT assays were then performed. ^*^p < 0.05; ^**^p < 0.005. **(C)** PC-9/ER cells were treated without or with CQ (50 μM) for 24 h, followed by immunoblot and immunoprecipitation. **(D)** PC-9/ER cells were treated various concentrations of CQ for 24 h, followed by immunoblot. **(E)** PC-9/ER cells were treated CQ (50 μM) for 24 h. Cells were then transfected with Flag-CAGE construct (1 μg) for 24 h, followed by immunoblot.

The effect of inhibition of autophagy on anti-cancer drug-resistance was examined. Chloroquine (CQ), an inhibitor of autophagy, induced the cleavage of PARP in response to erlotinib and osimertinib (Figure 3A) and enhanced the sensitivity of PC-9/ER cells to erlotinib and osimertinib (Figure 3B). CQ decreased expression levels of pBeclin1^Ser15^ and ATG5, but increased expression levels of LC3-II and p62 in PC-9/ER cells (Figure 3C). CQ inhibited the binding of CAGE to Beclin1 in PC-9/ER cells (Figure 3C) and decreased the expression of CAGE in PC-9/ER cells in a dose-dependent manner (Figure 3D). The binding of CAGE to Beclin1 suggests a role of CAGE in autophagic processes. CAGE can bind to HER2 in anti-cancer drug-resistant melanoma cells (27) while HER2 can bind to Beclin1 in breast cancer cells and inhibits autophagy (28). This led us to hypothesize that CAGE might bind to Beclin1. Reintroduction of CAGE increased expression levels of pBeclin1^Ser15^ and ATG5 in PC-9/ER cells treated with CQ (Figure 3E), suggesting that CQ could target CAGE. Thus, CAGE may regulate anti-cancer drug-resistance by binding to Beclin1. The binding of CAGE to Beclin1, a mediator of autophagy, in non-small cell lung cancer cells has not been reported. Further studies are needed to identify targets of CAGE for better understanding of the enhanced sensitivity to anti-cancer drugs conferred by CQ.

### CAGE Binding to Beclin1 Occurs in Anti-cancer Drug-Resistant Cancer Cells and Serves as a Target of CAGE-Derive Peptide

**Figure 4:**
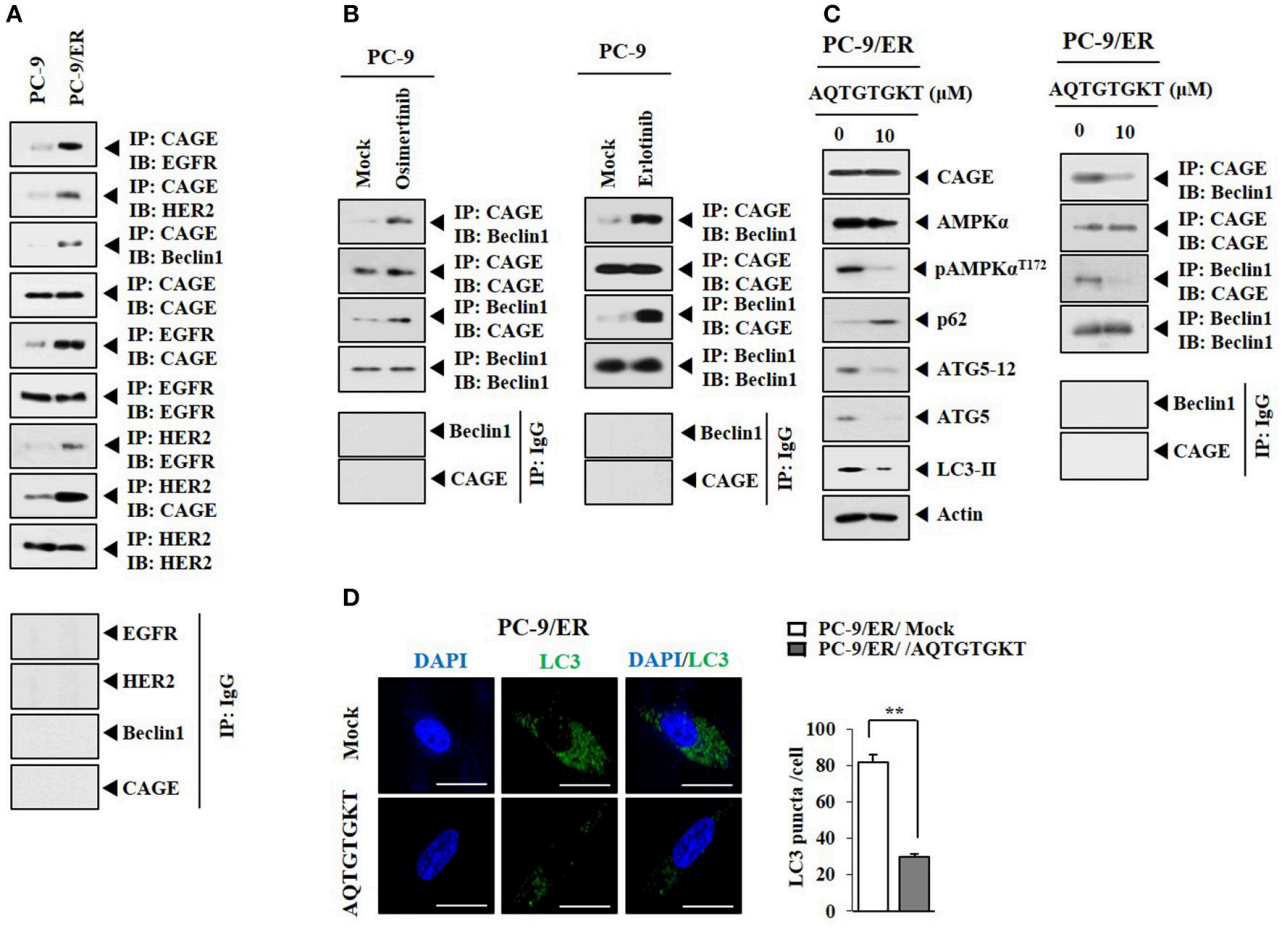
CAGE binding to Beclin1 occurs in anti-cancer drug-resistant cancer cells and serves as a target of CAGE-derive peptide. **(A)** Cell lysates from the indicated cancer cells were subjected to immunoblot and immunoprecipitation. **(B)** PC-9 cells were treated without or with erlotinib (20 μM) or osimertinib (5 μM) for 24 h followed by immunoprecipitation. **(C)** PC-9/ER cells were transfected with AQTGTGKT peptide (10 μM) for 24 h followed by immunoblot and immunoprecipitation. **(D)** PC-9/ER cells were transfected with AQTGTGKT peptide (10 μM) for 24 h followed by immunofluorescence staining. ^**^p < 0.005.

**Figure 5:**
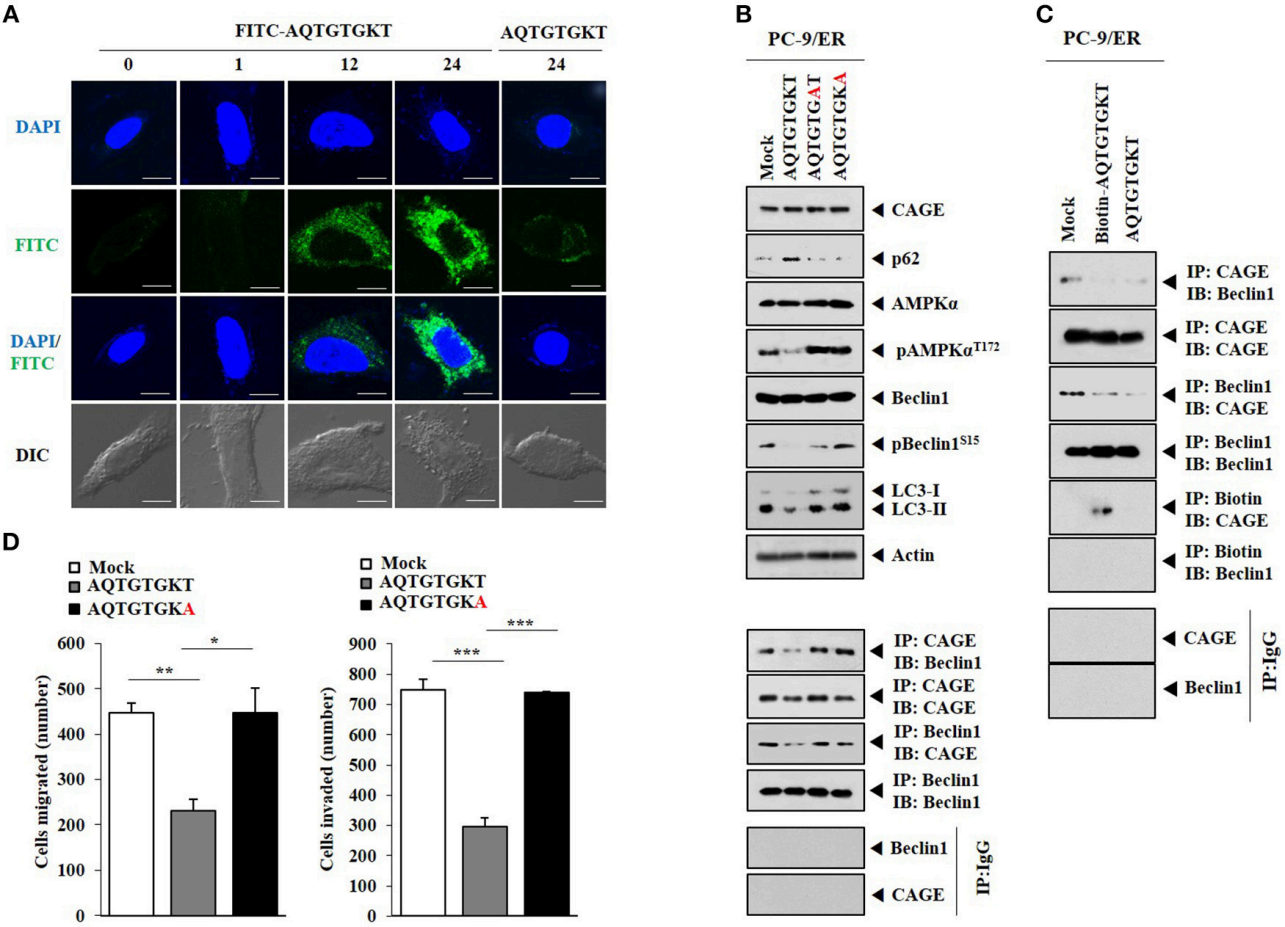
AQTGTGKT peptide, but not mutant peptides, binds to CAGE and regulates autophagc flux and the binding of CAGE to Beclin1. **(A)** PC-9/ER cells were transfected with FITC-AQTGTGKT peptide (10 μM) or unlabeled AQTGTGKT peptide (10 μM). At each time point after transfection, fluorescence microscopic observation was performed. Scale bar represents 10 μm. **(B)** PC-9/ER cells were transfected with the indicated peptide (each at 10 μM). At 24 h after transfection, immunoblot, and immunoprecipitation were performed. **(C)** PC-9/ER cells were transfected with the indicated peptide (each at 10 μM). At 24 h after transfection, immunoprecipitation was performed. **(D)** Same as **(C)** except that migration and invasion assays were performed. ^*^p < 0.05; ^**^p < 0.005; ^***^p < 0.0005.

HER2 showed binding to Beclin1 in PC-9/ER cells (Figure 4A). CAGE showed binding to Beclin1, EGFR, and HER2 in PC-9/ER cells, but not in PC-9 cells (Figure 4A). Rapamycin induced binding of CAGE to Beclin1 in PC-9 cells (Supplementary Figure 3). Erlotinib and osimertinib also induced binding of CAGE to Beclin1 in PC-9 cells (Figure 4B). CAGE-derived peptides such as ^269^GTGKT^273^ and ^266^AQTGTGKT^273^ can overcome anti-cancer drug-resistance in melanoma cells (11). Peptide ^266^AQTGTGKT^273^ corresponds to DEAD box domain of CAGE. AQTGTGKT peptide decreased expression levels of pAMPKα^Thr172^, ATG5, and LC3-II and prevented CAGE from binding to Beclin1 in PC-9/ER cells (Figure 4C). However, AQTGTGKT peptide did not affect the expression of CAGE (Figure 4C). AQTGTGKT peptide decreased LC3 puncta expression in PC-9/ER cells (Figure 4D). The uptake of AQTGTGKT peptide into PC-9/ER cells was confirmed by employing FITC-AQTGTGKT peptide (Figure 5A). AQTGTGKT peptide, but not mutant AQTGTGAT or AQTGTGKA peptide, regulated autophagic flux and inhibited the binding of CAGE to Beclin1 (Figure 5B). We also examined the possibility of binding of AQTGTGKT peptide to CAGE or Beclin1. For this, we employed biotin-AQTGTGKT peptide. AQTGTGKT peptide showed binding to CAGE, but not to Beclin1 in PC-9/ER cells (Figure 5C). AQTGTGKT peptide and biotin-AQTGTGKT peptide inhibited binding of CAGE to Beclin1 in PC-9/ER cells (Figure 5C). AQTGTGKT peptide, but not mutant AQTGTGKA peptide, decreased the migration and invasion potential of PC-9/ER cells (Figure 5D). These results suggest that CAGE binding to Beclin1 can serve as a target for CAGE-der*i*ved peptide to develop anti-cancer drugs. Thus, CAGE-derived AQTGTGKT peptide in combination with erlotinib or osimertinib may overcome resistance of non-small cell lung cancer cells to anti-cancer drugs.

### HER2 Regulates Responses to Anti-cancer Drugs

**Figure 6:**
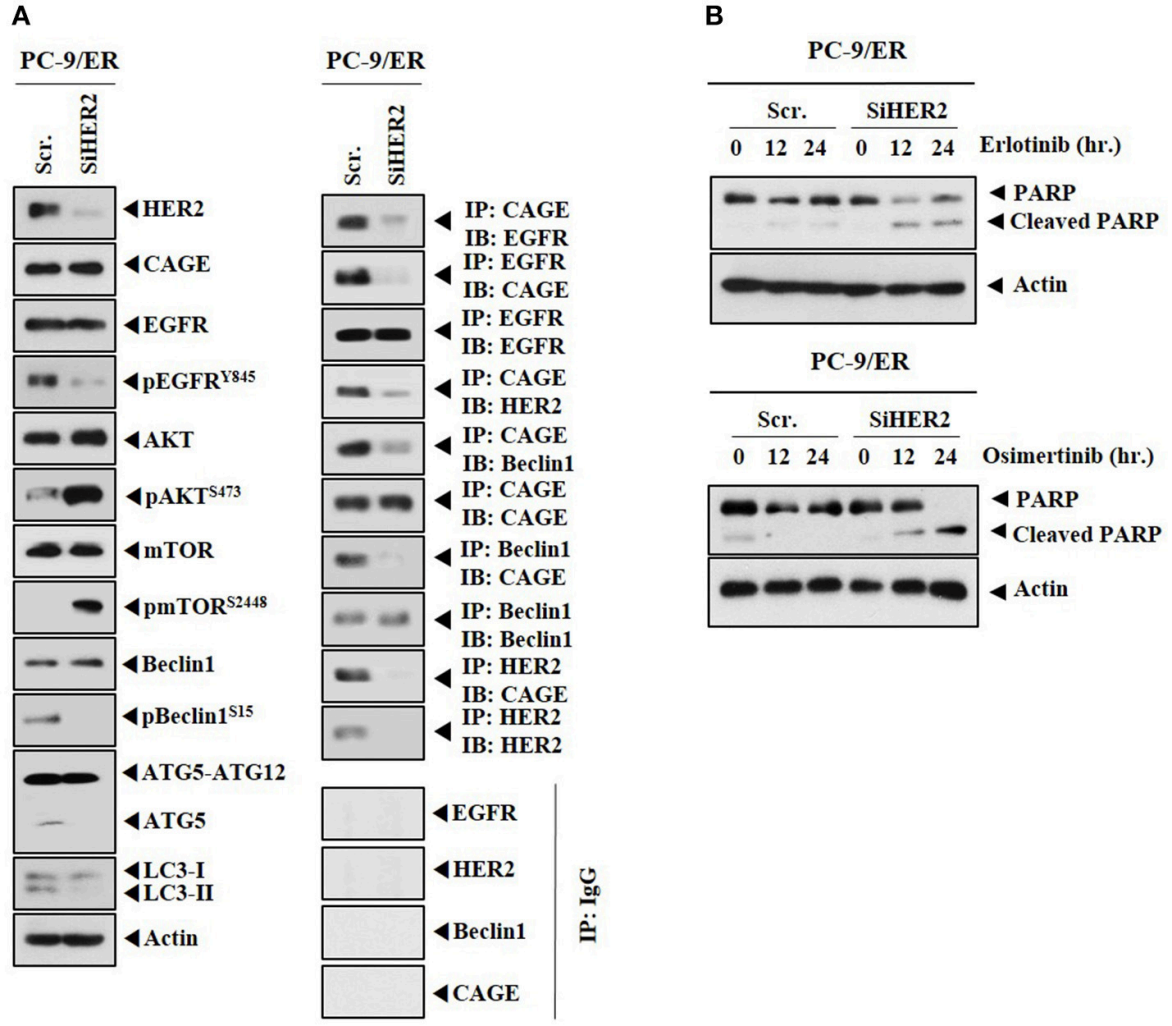
Down-regulation of HER2 enhances the sensitivity of PC-9/ER cells to anti-cancer drugs. **(A)** PC-9/ER cells were transfected with the indicated siRNA (each at 10 nM). At 48 h after transfection, immunoblot and immunoprecipitation were performed. **(B)** PC-9/ER cells were transfected with the indicated siRNA (each at 10 nM). On the next day, cells were then treated with the indicated anti-cancer drugs for various time intervals, followed by immunoblot.

CAGE showed binding to HER2 in PC-9/ER (Figure 4A). The role of HER2 in anti-cancer drug-resistance was therefore examined. Down-regulation of HER2 decreased autophagic flux, but increased expression levels of pAKT^Ser473^ and pmTOR^Ser2448^ in PC-9/ER cells (Figure 6A). Down-regulation of HER2 decreased the expression of pEGFR^Y845^, but not the expression of CAGE in PC-9/ER cells (Figure 6A). HER2 was necessary for the binding of CAGE to Beclin1 and EGFR (Figure 6A). Down-regulation of HER2 increased cleavage of PARP in response to erlotinib and osimertinib in PC-9/ER cells (Figure 6B). Thus, HER2 is necessary for binding of CAGE to Beclin1 and regulates responses to anti-cancer drugs.

### CAGE-Derived AQTGTGKT Peptide Confers Sensitivity to Anti-cancer Drugs

**Figure 7:**
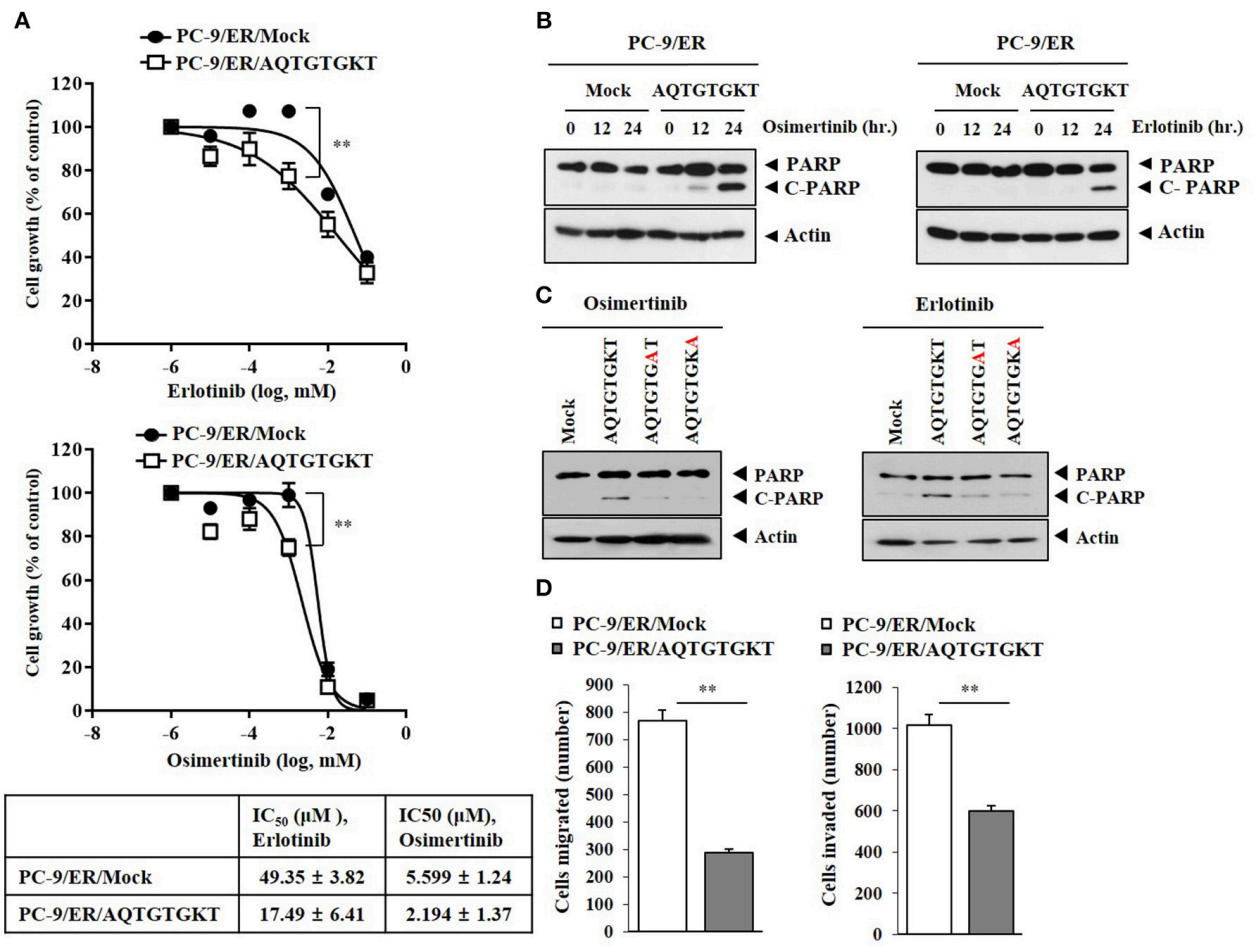
CAGE-derived peptide confers sensitivity to anti-cancer drugs. **(A)** PC-9/ER cells were transfected with AQTGTGKT peptide (10 μM) for 24 h followed by treatment with various concentrations of the indicated anti-cancer drugs for 24 h. ^**^p < 0.005. **(B)** PC-9/ER cells were transfected with AQTGTGKT peptide (10 μM) for 24 h followed by treatment with erlotinib (20 μM) or osimertinib (5 μM) for various time intervals. Immunoblot was then performed. **(C)** PC-9/ER cells were transfected with the indicated peptide (each at 10 μM) for 24 h followed by treatment with erlotinib (20 μM) or osimertinib (5 μM) for 24 h. Immunoblot was then performed. **(D)** PC-9/ER cells were transfected with AQTGTGKT peptide (10 μM) for 24 h followed by migration and invasion analysis. ^**^p < 0.005.

Because AQTGTGKT peptide decreased autophagic flux and inhibited the binding of CAGE to Beclin1 (Figure 4C), the effect of AQTGTGKT peptide on anti-cancer drug-resistance was examined. AQTGTGKT peptide enhanced the sensitivity of PC-9/ER cells to erlotinib and osimertinib (Figure 7A) and the cleavage of PARP in response to erlotinib and osimertinib (Figure 7B). AQTGTGKT peptide, but not mutant AQTGTGAT or AQTGTGKA peptide, enhanced the cleavage of PARP in response to erlotinib and osimertinib (Figure 7C). AQTGTGKT peptide decreased the migration and invasion potential of PC-9/ER cells (Figure 7D). The effect of AQTGTGKT peptide on autophagic flux in H1975 non-small cell lung cancer cells with EGFR mutations (L885R/T790 M) was examined. AQTGTGKT peptide decreased expression levels of pBeclin1^Ser15^, pAMPKα^Thr172^, LC3-II, and ATG5, but increased expression levels of p62 and pmTOR^Ser2448^ (Supplementary Figure 4A). AQTGTGKT peptide inhibited binding of CAGE to Beclin1 (Supplementary Figure 4B) and enhanced the cleavage of PARP in response to anti-cancer drugs (Supplementary Figure 4C). AQTGTGKT peptide decreased the migration and invasion potential of H1975 cells (Supplementary Figure 4D) and enhanced the sensitivity of H1975 cells to anti-cancer drugs (Supplementary Figure 4E). AQTGTGKT peptide decreased LC3 puncta expression in H1975 cells (Supplementary Figure 4F). AQTGTGKT peptide, but not AQTGTGAT or AQTGTGKA, regulated autophagic flux in H1975 cells (Supplementary Figure 4G). The uptake of AQTGTGKT peptide into H1975 cells was examined using FITC-AQTGTGKT peptide (Supplementary Figure 5A). AQTGTGKT peptide, but not AQTGTGAT or AQTGTGKA, inhibited the binding of CAGE to Beclin1 H1975 cells (Supplementary Figure 5B). Biotin-AQTGTGKT peptide, just like AQTGTGKT peptide, inhibited the binding of CAGE to Beclin1 in H1975 cells (Supplementary Figure 5C). Biotin-AQTGTGKT peptide showed binding to CAGE, but not to Beclin1 in H1975 cells (Supplementary Figure 5C). CAGE showed nuclear and cytoplasmic localization in PC-9/ER cells and co-localization with AQTGTGKT peptide in the cytoplasm in PC-9/ER cells (Supplementary Figure 6A). AQTGTGKT peptide, but not AQTGTGAT or AQTGTGKA, enhanced the cleavage of PARP in response to erlotinib and osimertinib in H1975 cells (Supplementary Figure 6B). Our results provide evidence that AQTGTGKT peptide is a potential anti-cancer drug for the treatment of non-small cell lung cancers.

### CAGE Regulates Autophagic Flux and Anti-cancer Drug-Resistance

Because CAGE showed binding to Beclin1 in PC-9/ER cells (Figure 4A), the effect of CAGE on autophagic flux was examined. Down-regulation of CAGE decreased expression levels of pBeclin1^Ser15^, pAMPKα^Thr172^, pEGFR^Y845^, and pHER2^Y1248^, but increased expression levels of pmTOR^Ser2448^ and p62 in PC-9/ER cells (Supplementary Figure 7A). Since CAGE showed binding to EGFR and HER2 (Figure 4A), CAGE might be necessary for the activation of EGFR and HER2 in PC-9/ER cells. Down-regulation of CAGE enhanced the cleavage of PARP in response to erlotinib and osimertinib in PC-9/ER cells (Supplementary Figure 7B), decreased expression levels of pBeclin1^Ser15^, ATG5-12, pAMPKα^Thr172^, and LC3-II in H1975 cells (Supplementary Figure 7C), and increased expression of pmTOR^Ser2448^ (Supplementary Figure 7C). Down-regulation of CAGE resulted in disruption of CAGE-Beclin1 interaction (Supplementary Figure 7C). It also enhanced the cleavage of PARP in response to erlotinib and osimertinib in H1975 cells (Supplementary Figure 7D) and decreased the migration and invasion potential of H1975 cells (Supplementary Figure 7E). Thus, CAGE can regulate anti-cancer drug-resistance by regulating autophagic flux in PC-9/ER cells and H1975 cells.

### CAGE-Derived Peptide Decreases Tumorigenic Potential of Anti-cancer Drug-Resistant Non-small cell Lung Cancer Cells

**Figure 8:**
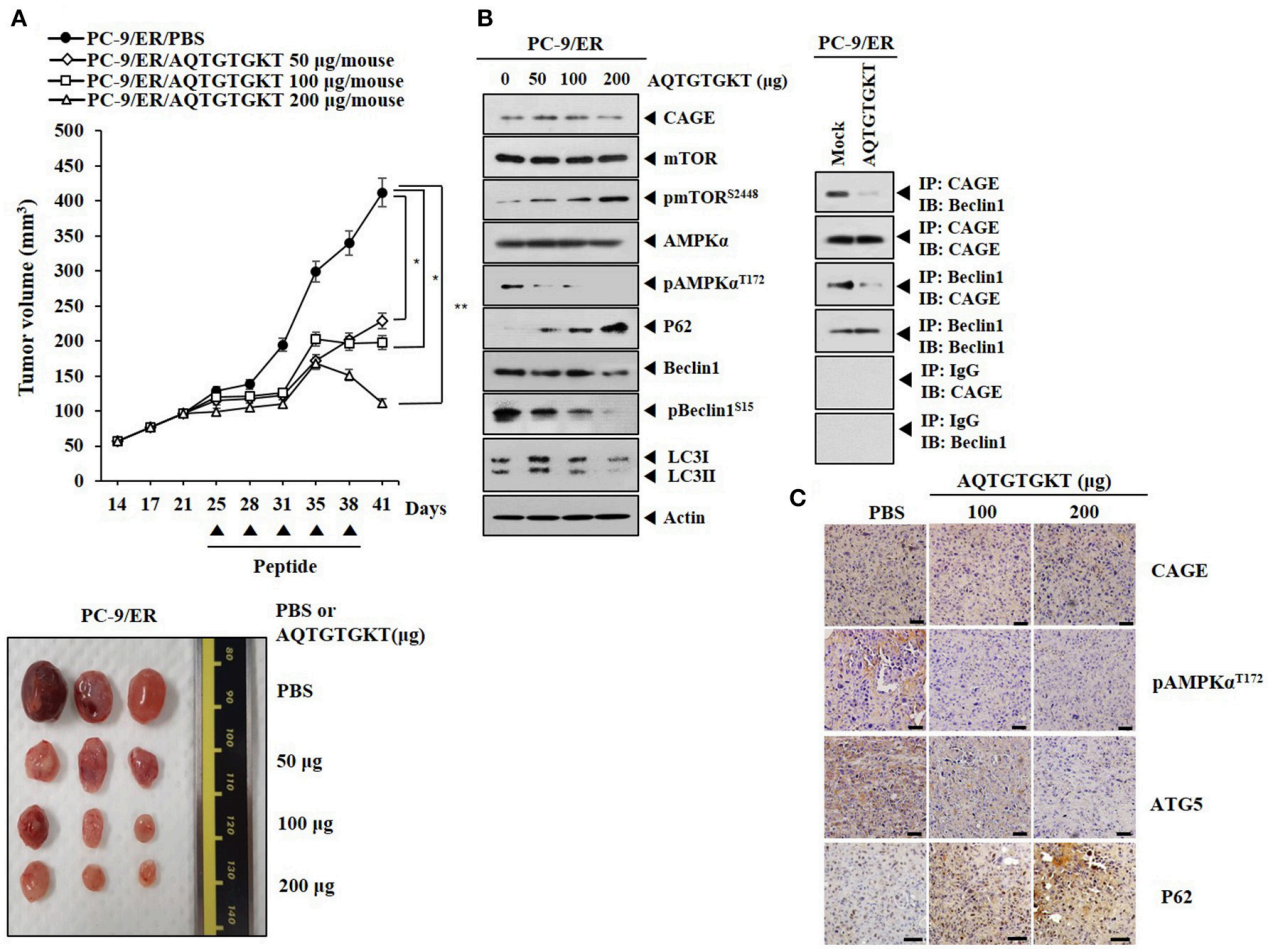
**A**QTGTGKT peptide decreases tumorigenic potential of PC-9/ER cells. **(A)** PC-9/ER (1 × 10^6^) cells were injected into dorsal flanks of athymic nude mice. Following the establishment of sizeable tumor (~50 mm^3^), AQTGTGKT peptide (50, 100, 200 μg/mouse) was injected into the tail vein five times in a total of 41 days. Each experimental group consisted of five mice. Each value represents an average obtained from the five athymic nude mice of each group. Data are expressed as a mean ± S.D. Each figure shows a representative image of mouse in each group at the time of sacrifice. ^*^p < 0.05; ^**^p < 0.005. **(B)** Lysates from the indicated tumor tissues were subjected to immunoblot and immunoprecipitation employing the indicated antibody (2 μg/ml). **(C)** Immunohistochemical staining of tumor tissues employing the indicated antibody (2 μg/ml) was performed. Scale bar represents 100 μm.

We examined the effect of AQTGTGKT peptide on the tumorigenic potential of PC-9/ER cells. AQTGTGKT peptide decreased the tumorigenic potential of PC-9/ER cells (Figure 8A). AQTGTGKT peptide decreased expression levels of pBeclin1^Ser15^ and pAMPKα^Thr172^, but increased expression levels of p62 and pmTOR^Ser2448^ (Figure 8B). Immunoprecipitation showed that AQTGTGKT peptide inhibited binding of CAGE to Beclin1 (Figure 8B). Immunohistochemical staining employing tumor tissues showed that AQTGTGKT peptide decreased expression levels of ATG5 and pAMPKα^Thr172^, but not CAGE (Figure 8C). Immunohistochemical staining also showed that AQTGTGKT peptide increased expression level of p62 (Figure 8C). We next examined the effect of AQTGTGKT peptide on tumorigenic potential of H1975 cells. AQTGTGKT peptide decreased tumorigenic potential of H1975 cells (Supplementary Figure 8A). AQTGTGKT peptide regulated autophagic flux (Supplementary Figure 8B) and inhibited binding of CAGE to Beclin1 (Supplementary Figure 8B). Immunohistochemical staining showed that AQTGTGKT peptide decreased expression levels of ATG5 and pAMPKα^Thr172^, but not CAGE (Supplementary Figure 8C). Immunohistochemical staining also showed that AQTGTGKT peptide increased expression level of p62 (Supplementary Figure 8C). Thus, AQTGTGKT peptide can decrease tumorigenic potential of PC-9/ER cells and H1975 cells by regulating autophagic flux and binding of CAGE to Beclin1.

### CAGE-Derived Peptide Shows *Ex vivo* Tumor Homing Potential

**Figure 9:**
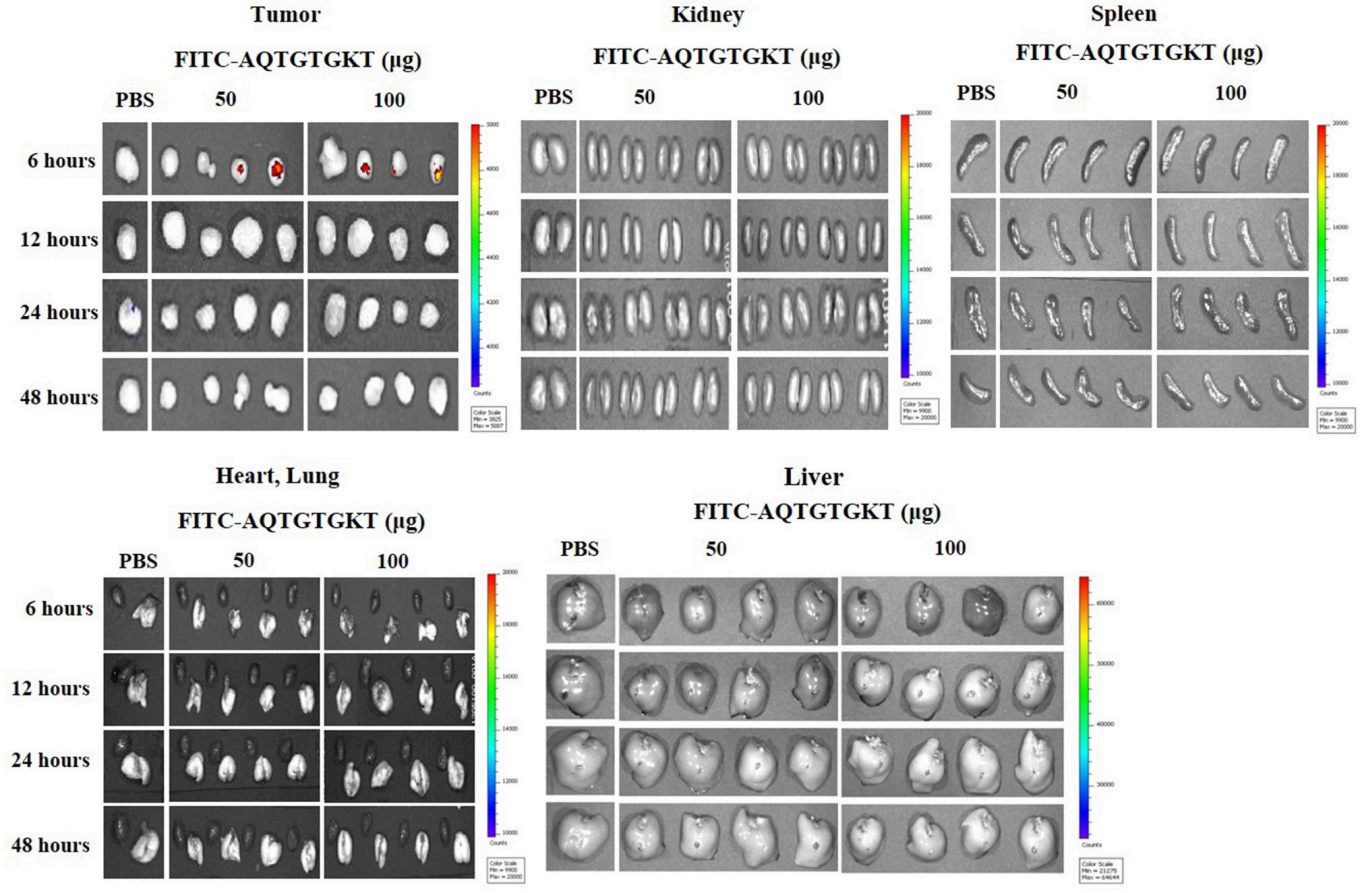
AQTGTGKT peptide shows *ex vivo* tumor homing potential. Tumor-bearing nude mice (xenograft of H1975 cells) were given intravenous injection of FITC-conjugated AQTGTGKT peptide after tumors reached a certain size (200–300 mm^3^). Representative distribution of FITC-conjugated AQTGTGKT peptide at the indicated time after injection in dissected tumor and normal organs including kidney, liver, spleen, and lung is shown. Note the strong fluorescence accumulation in the tumor compared to that in control organs.

Because AQTGTGKT peptide decreased tumorigenic potentials of PC-9/ER cells (Figure 8A) and H1975 cells (Supplementary Figure 8C), we examined whether AQTGTGKT peptide could display tumor homing potential. To determine distribution of the FITC-conjugated AQTGTGKT peptide in tumor bearing animals and examine whether FITC-conjugated AQTGTGKT peptide was localized to tumor *in vivo*, tumor xenografts (H1975 cells) were injected intravenously with FITC-conjugated AQTGTGKT peptide. The peptide was then allowed to circulate in the blood stream for various time intervals before tumors and control organs were excised. Strong and specific fluorescence was detected in tumor xenografts (H1975 cells) at 6 h after injection with FITC-conjugated AQTGTGKT peptide. However, little labeling was seen in control organs (Figure 9). Therefore, AQTGTGKT peptide shows tumor homing to decrease tumorigenic potential of H1975 cells.

### AQTGTGKT Peptide Increases Expression Levels of miR-143-3p and miR-373-5p in PC-9/ER Cells and Confers Sensitivity to Anti-cancer Drugs

**Figure 10:**
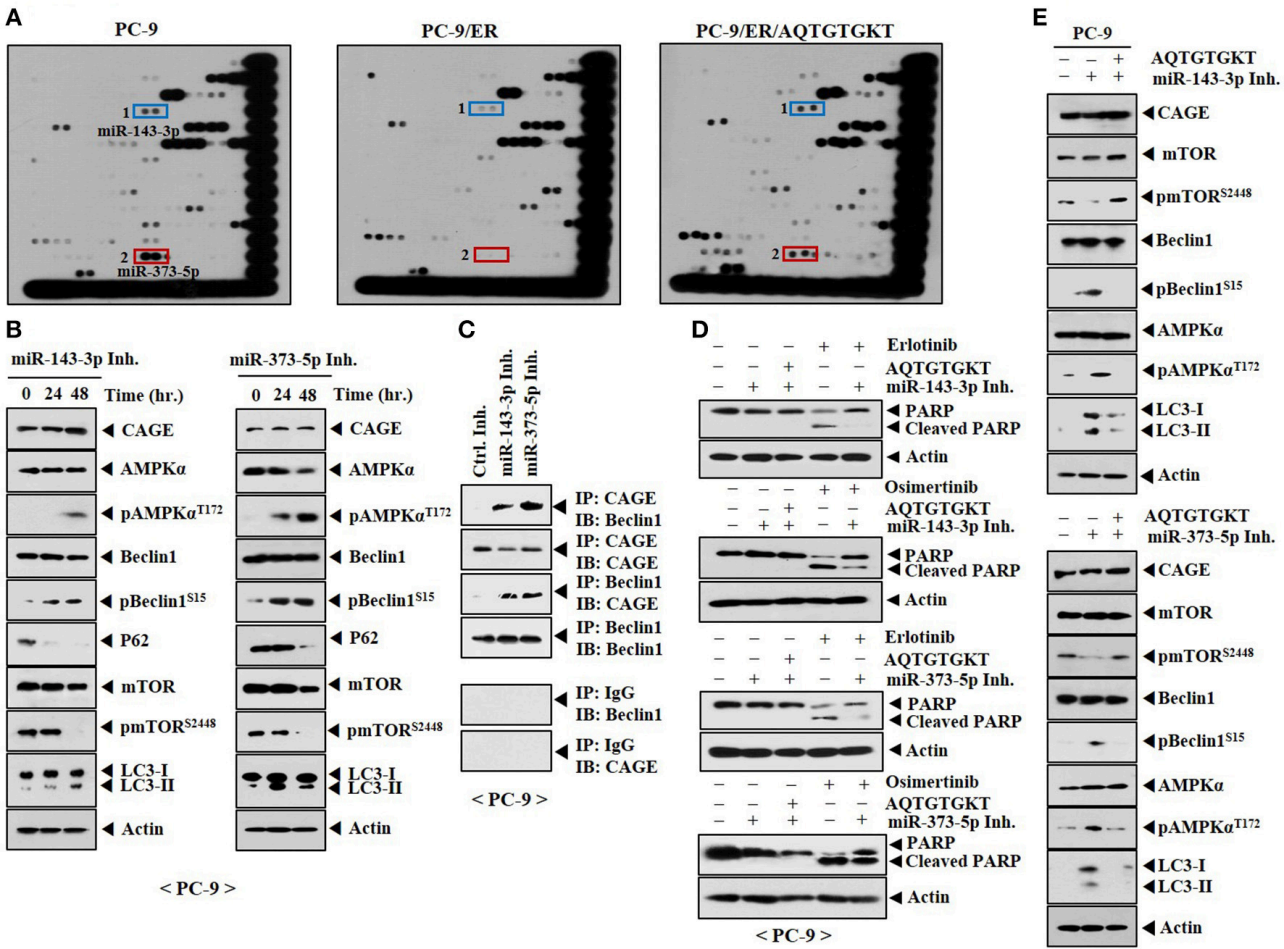
AQTGTGKT peptide increases the expression of miR-143-3p and miR-373-5p to confer sensitivity to anti-cancer drugs. **(A)** Total RNAs isolated from the indicated cancer cells were subjected to miRNA array analysis. PC-9/ER cells were transfected without or with AQTGTGKT (10 μM) for 24 h. **(B)** PC-9 cells were transfected with the indicated inhibitor (each at 10 nM). At each time point after transfection, cell lysates were isolated and subjected to immunoblot. **(C)** PC-9 cells were transfected with the indicated inhibitor (each at 10 nM) for 48 h, followed by immunoprecipitation. **(D)** PC-9 cells were transfected with the indicated inhibitor without or with AQTGTGKT peptide. On the next day, cells were then treated without or with erlotinib (20 μM) or osimertinib (5 μM) for 24 h, followed by immunoblot. **(E)** PC-9 cells were transfected with the indicated inhibitor (each at 10 nM) without or with AQTGTGKT peptide (10 μM). At 48 h after transfection, immunoblot was performed.

To better understand the mechanism involved in the resistance of non-small cell lung cancer cells to anti-cancer drugs, miRNA array analysis was performed. PC-9 cells showed higher expression levels of miR-143-3p and miR-373-5p than PC-9/ER cells (Figure 10A). Expression levels of miR-143-3p and miR-373-5p were restored by AQTGTGKT peptide in PC-9/ER cells (Figure 10A). Overexpression of miR-143-3p inhibitor or miR-373-5p inhibitor increased autophagic flux, but decreased expression levels of p62 and pmTOR^Ser2448^ in PC-9 cells (Figure 10B). MiR-143-3p inhibitor and miR-373-5p inhibitor induced the binding of CAGE to Beclin1 in PC-9 cells (Figure 10C). MiR-143-3p inhibitor and miR-373-5p inhibitor negatively regulated the effect of erlotinib and osimertinib on the cleavage of PARP in PC-9 cells (Figure 10D). AQTGTGKT peptide prevented miR-143-3p inhibitor and miR-373-5p inhibitor from increasing autophagic fluxin PC-9 cells (Figure 10E). Thus, targets of AQTGTGKT peptide such as miR-143-3p and miR-373-5p can regulate autophagic flux and the sensitivity to anti-cancer drugs.

## Discussion

Induction of autophagy is associated with anti-cancer drug-resistance (29). Inhibition of autophagy could sensitize cancer cells to 5-FU-induced caspase-dependent apoptosis through stimulation of ROS formation (23). Erlotinib treatment at clinically relevant concentrations can induce autophagy in sensitive non-small cell lung cancer cell lines via p53 nuclear translocation, AMPK activation, and mTOR suppression (30). Our results revealed that PC-9/ER cells had higher levels of autophagic flux than PC-9 parental anti-cancer drug-sensitive non-small cell lung cancer cells (Figure 1C). Our results also showed that erlotinib and osimertinib increased expression of pAMPKα^Thr172^, but decreased expression of pmTOR^Ser2448^ in PC-9 cells. This suggests that resistance to erlotinib and osimertinib results from autophagy induced by these anti-cancer drugs. We showed that rapamycin and CQ could regulate responses to erlotinib and osimertinib. Our results established a close relationship between anti-cancer drug-resistance and autophagy. Identification of targets of rapamycin and CQ will provide clear understanding of anti-cancer drug-resistance in non-small cell lung cancer cells. Further studies are needed to examine whether CQ could induce ubiquitination of CAGE to decrease the expression of CAGE.

Our results showed binding of CAGE to Beclin1 in PC-9/ER cells. Further study is needed to determine the domain of CAGE necessary for binding to Beclin1. We have shown the importance of DEAD box domain of CAGE in conferring resistance to anti-cancer drugs in melanoma cells (11). Thus, DEAD box domain of CAGE may be necessary for the binding of CAGE to Beclin1. Because CAGE binds to Beclin1, it will be interesting to examine whether CAGE displays kinase activity by employing recombinant CAGE protein. CAGE binds to GSK3ß in anti-cancer drug-resistant melanoma cells (11). We showed that CAGE-derived peptide such as AQTGTGKT could inhibit binding of CAGE to Beclin1 and conferred confer sensitivity of PC-9/ER cells to anti-cancer drugs. We identified essential residues within AQTGTGKT peptide for inhibiting the binding of CAGE to Beclin1. CAGE-Beclin1 interaction leads to autophagy and confers resistance to anti-cancer drugs in non-small cell lung cancer cells. We found that AQTGTGKT peptide showed binding to CAGE, but not to Beclin1. It would be necessary to determine the domain of CAGE that binds to AQTGTGKT peptide.

PC-9/ER cells are known to have higher invasion and migration potential than PC-9 cells (19). We showed that AQTGTGKT peptide decreased the migration and invasion potential of PC-9/ER cells. It is probable that AQTGTGKT peptide might be able to decrease the expression of SNAIL while increasing the expression of E-cadherin to affect the migration and invasion potential of PC-9/ER cells. Increased MET activity is associated with resistance to erlotinib in PC-9/ER cells (19) and cell migration (31). It will be necessary to examine the effect of AQTGTGKT peptide on MET activity in PC-9/ER cells in the future.

Down-regulation of Beclin1 decreases expression of HER2, contributes to alteration of tamoxifen sensitivity and predicts favorable outcome in ER-positive breast cancer (32). We showed that down-regulation of HER2 also decreased the expression of pEGFR^Y845^ and pBeclin1^Ser15^ in PC-9/ER cells (Figure 6A). Down-regulation of HER2 enhanced the sensitivity to anti-cancer drugs in PC-9/ER cells (Figure 6B). These results suggest that CAGE-HER2 interaction is necessary for the increased expression of pEGFR^Y845^ and pBeclin1^Ser15^ in PC-9/ER cells.

Resistance to HER2-targeted anti-cancer drugs such as trastuzumab is associated with extracellular vesicles (33). Exosomes derived from gefitinib-treated PC-9 cells can decrease the antitumor effects of cisplatin by upregulating autophagic flux (34). Exosomes from PC-9/ER cells might confer resistance to anti-cancer drugs in PC-9 cells. Further study is needed to examine whether CAGE is within the exosomes of PC-9/ER cells. In addition, molecules differentially expressed between exosomes of PC-9 cells and PC-9/ER cells need to be identified in the future.

Previous reports have suggested roles of miRNAs in anti-cancer drug-resistance (7, 11). We showed that expression levels of miR-143-3p and miR-373-5p were lower in PC-9/ER cells than those in PC-9 cells while AQTGTGKT peptide could restore expression levels of miR-143-3p and miR-373-5p in PC-9/ER cells. This implies that miR-143-3p and miR-373-5p may decrease autophagic flux in PC-9/ER cells and confer sensitivity to anti-cancer drugs. We showed that miR-143-3p inhibitor and miR-373-5p inhibitor increased autophagic flux in PC-9 cells and induced the binding of CAGE to Beclin1 in PC-9 cells. It would be interesting to examine factor(s) that may prevent the binding of CAGE to Beclin1 in PC-9 cells. Further study is needed to examine effects of miR-143-3p and miR-373-5p on the tumorigenic potentials of PC-9/ER cells and H1975 cells. It will be also interesting to examine whether miR-143-3p and miR-373-5p are differentially expressed between PC-9 and PC-9/ER cells. Human miR-143-3p can suppress tumor angiogenesis and growth of gall bladder carcinoma through ITGA6/PI3K/AKT/PLGF pathways (35). Human miR-143-3p is known to act as a novel tumor suppressive miRNA by regulating tumor growth, migration and invasion through directly targeting AKT2 gene (36).

TargetScan analysis predicted the binding of miR-143-3p to the 3′-UTR of KRAS, ATG10, FOXO1, and FGF1 (personal observations). KRAS-RalB-NF-κB pathway was both necessary and sufficient for tumor initiation, anchorage independence, self-renewal, and erlotinib resistance (37). ATG10 is necessary for lung cancer survival (38). MiR-143-3p promoter sequences contain binding sites for AP1, HDAC2, P53, Elk-1, YY1, and DNMT1 (personal observations). CAGE binds to HDAC2 and confers resistance to anti-cancer drugs in melanoma cells (9). This suggests that CAGE may regulate expression of miR-143-3p. MiR-373-5p promoter sequences contain binding sites for C-Jun, AP1, P53, Elk-1, YY1, GATA1, and STAT4 (personal observations). TargetScan analysis predicted binding of miR-373-5p to the 3′-UTR of HIF1-α, STAT1, and ATG12 (personal observations). Low dose erlotinib-cisplatin combination exhibits its anti-tumor activity by targeting angiogenesis through modulation of c-MYC/HIF-1α/VEGF pathway in non-small cell lung cancer cells with EGFR exon 19 deletions (39). The outgrowth of trastuzumab-unresponsive tumors is completely prevented when trastuzumab treatment is administered in an ATG12-silenced genetic background (40).

In summary, we showed novel roles of CAGE and CAGE-derived peptide in responses of non-small cell lung cancer cells to anti-cancer drugs. We provided evidence that combination of anti-cancer drug such as erlotinib or osimertinib with CAGE-derived peptide could overcome resistance of non-small cell lung cancers to anti-cancer drugs.

## Author Contributions

DooJ designed the study and analyzed the data. MY, JB, MK, HK, DoyJ and YK performed experiments. DooJ and HJ planned the projects and supervised the experiments. DooJ wrote the manuscript.

### Conflict of Interest Statement

The authors declare that the research was conducted in the absence of any commercial or financial relationships that could be construed as a potential conflict of interest.

## Supplementary Material

Supplementary Figure 1 P62, an autophagic receptor, regulates autophagic flux. PC-9 cells were transfected with indicated siRNA (each at 10 nM). At 48 h after transfection, immunoblot was performed. Scr. denotes scrambled siRNA.

Supplementary Figure 2 Rapamycin confers resistance to anti-cancer drugs and increases autophagic flux. **(A)** PC-9 cells were treated with rapamycin (20 μM) for various time intervals. Cell lysates isolated at each time point were subjected to immunoblot. **(B)** PC-9 cells were treated without or with rapamyicn (20 μM) for 24 h, followed by LC3 puncta staining. Scale bar represents 10 μm. Immunofluorescence staining using only secondary antibody was also performed as negative control. ^**^*p* < 0.005. **(C)** PC-9 cells were treated without or with rapamycin (20 μM) for 24 h, followed by treatment with erlotinib (20 μM) or osimertinib (5 μM) for 24 h.

Supplementary Figure 3 Rapamycin induces binding of CAGE to Beclin1. PC-9 cells were treated without or with rapamycin (20 μM) for 24 h, followed by immunoprecipitation.

Supplementary Figure 4 AQTGTGKT peptide confers sensitivity to anti-cancer drugs by regulating autophagic flux in non-small cell lung cancer cells with EGFR mutation (L885R/T790 M). **(A)** H1975 cells were transfected with AQTGTGKT peptide (10 μM) for 24 h followed by immunoblot. **(B)** Same as **(A)** except that immunoprecipitation was performed. **(C)** H1975 cells were transfected with AQTGTGKT peptide (10 μM) for 24 h followed by treatment with erlotinib (20 μM) or osimertinib (5 μM) for various time intervals. Immunoblot was then performed. **(D)** H1975 cells were transfected with AQTGTGKT peptide (10 μM) for 48 h followed by migration and invasion assays. ^*^*p* < 0.05; ^**^*p* < 0.005. **(E)** H1975 cells were transfected with AQTGTGKT peptide (10 μM) for 24 h followed by treatment with various concentrations of erlotinib or osimertinib for 24 h. MTT assays were then performed. **(F)** Same as **(A)** except that immunofluorescence staining was performed. ^***^*p* < 0.0005. **(G)** H1975 cells were transfected with the indicated peptide (10 μM) for 24 h followed by immunoblot.

Supplementary Figure 5 AQTGTGKT peptide binds to CAGE and inhibits the binding of CAGE to Beclin1. **(A)** H1975 cells were transfected with FITC-AQTGTGKT peptide (10 μM) or unlabeled AQTGTGKT peptide (10 μM). At each time point after transfection, fluorescence microscopic observation was performed. Scale bar represents 10 μm. **(B)** H1975 cells were transfected with wild type or mutant CAGE-derived peptide (each at 10 μM). At 24 h after transfection, immunoprecipitation was performed. **(C)** H1975 cells were transfected with the indicated peptide (10 μM). At 24 h after transfection, immunoprecipitation was performed.

Supplementary Figure 6 AQTGTGKT peptide shows co-localization with CAGE and enhances cleavage of PARP in response to erlotinib and osimertinib. **(A)** FITC-labeled AQTGTGKT peptide (10 μM) or unlabeled AQTGTGKT peptide (10 μM) was transfected into PC-9/ER cells. At 24 h after transfection, co-localization of AQTGTGKT peptide with CAGE was examined. Scale bar represents 10 μm. **(B)** H1975 cells were transfected with the indicated peptide (each at 10 μM). At 24 h after transfection, cells were then treated without or with erlotinib (20 μM) or osimertiniub (5 μM) for 24 h.

Supplementary Figure 7 CAGE regulates autophagic flux and anti-cancer drug-resistance. **(A)** PC-9/ER cell were transfected with the indicated siRNA (each at 10 nM). At 48 h after transfection, immunoblot, and immunoprecipitation were performed. **(B)** PC-9/ER cell were transfected with the indicated siRNA (each at 10 nM). On the next day, cells were then treated with erlotinib (20 μM) or osimertinib (5 μM) for various time intervals followed by immunoblot. **(C)** H1975 cells were transfected with the indicated siRNA (each at 10 nM). At 48 h after transfection, immunoblot, and immunoprecipitation were performed. **(D)** H1975 cells were transfected with the indicated siRNA (each at 10 nM). On the next day, cells were then treated with erlotinib (20 μM) or osimertinib (5 μM) for various time intervals followed by immunoblot. **(E)** H1975 cells were transfected with the indicated siRNA (each at 10 nM). At 48 h after transfection, migration, and invasion assays were performed. ^**^*p* < 0.005; ^***^*p* < 0.0005.

Supplementary Figure 8 AQTGTGKT peptide decreases tumorigenic potential of H1975 non-small cell lung cancer cells. **(A)** H1975 (1 × 10^6^) cells were injected into dorsal flanks of athymic nude mice. Following the establishment of sizeable tumor (~50 mm^3^), peptide (100, 200 μg/mouse) was injected into the tail vein five times in a total of 31 days. Each experimental group consisted of five mice. Each value represents an average obtained from the five athymic nude mice of each group. Data are expressed as a mean ± S.D. Each figure shows a representative image of mouse in each group at the time of sacrifice. ^**^*p* < 0.005. **(B)** Lysates from the indicated tumor tissues (200 μg/mouse) were subjected to immunoblot and immunoprecipitation. **(C)** Immunohistochemical staining of tumor tissues employing the indicated antibody (2 μg/ml) was performed. Scale bar represents 100 μm.
