# Supplementary figures and images for: CAGE Binds to Beclin1, Regulates Autophagic Flux and CAGE-Derived Peptide Confers Sensitivity to Anti-cancer Drugs in Non-small Cell Lung Cancer Cells
Source: Front Oncol. 2018 Dec 10;8:599. doi: 10.3389/fonc.2018.00599 (PMC6296237; doi:10.3389/fonc.2018.00599)

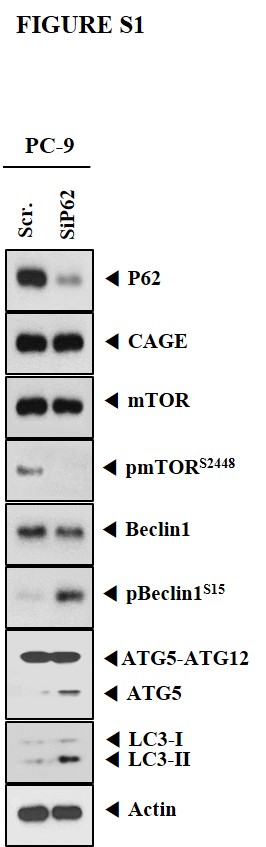

Supplement: Supplementary Figure 1 — P62, an autophagic receptor, regulates autophagic flux. PC-9 cells were transfected with indicated siRNA (each at 10 nM). At 48 h after transfection, immunoblot was performed. Scr. denotes scrambled siRNA. [file Image_1.JPEG]

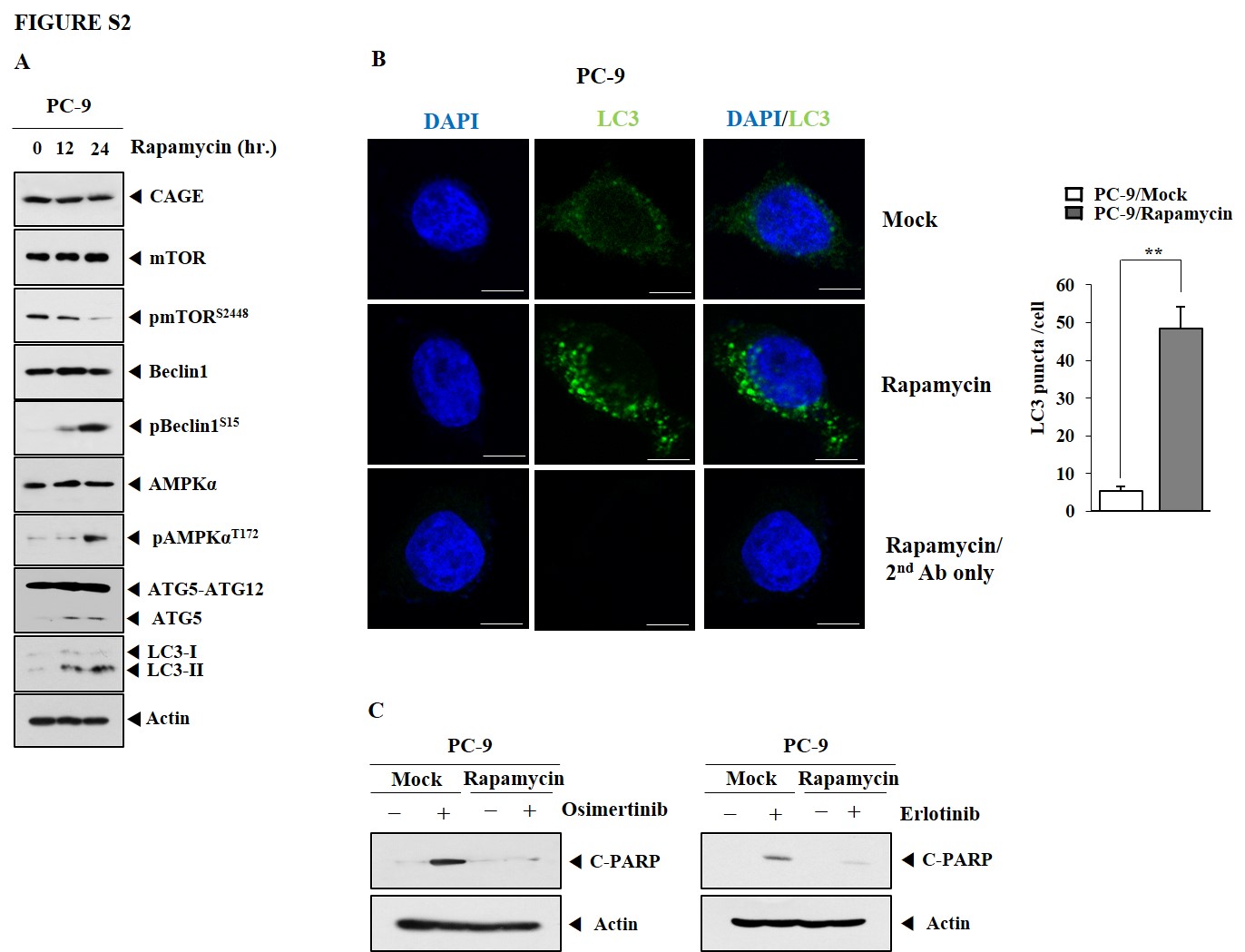

Supplement: Supplementary Figure 2 — Rapamycin confers resistance to anti-cancer drugs and increases autophagic flux. (A) PC-9 cells were treated with rapamycin (20 μM) for various time intervals. Cell lysates isolated at each time point were subjected to immunoblot. (B) PC-9 cells were treated without or with rapamyicn (20 μM) for 24 h, followed by LC3 puncta staining. Scale bar represents 10 μm. Immunofluorescence staining using only secondary antibody was also performed as negative control. **p < 0.005. (C) PC-9 cells were treated without or with rapamycin (20 μM) for 24 h, followed by treatment with erlotinib (20 μM) or osimertinib (5 μM) for 24 h. [file Image_2.JPEG]

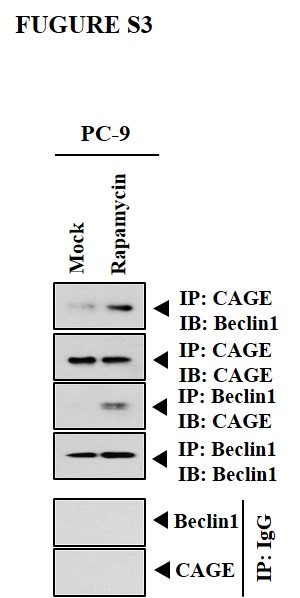

Supplement: Supplementary Figure 3 — Rapamycin induces binding of CAGE to Beclin1. PC-9 cells were treated without or with rapamycin (20 μM) for 24 h, followed by immunoprecipitation. [file Image_3.JPEG]

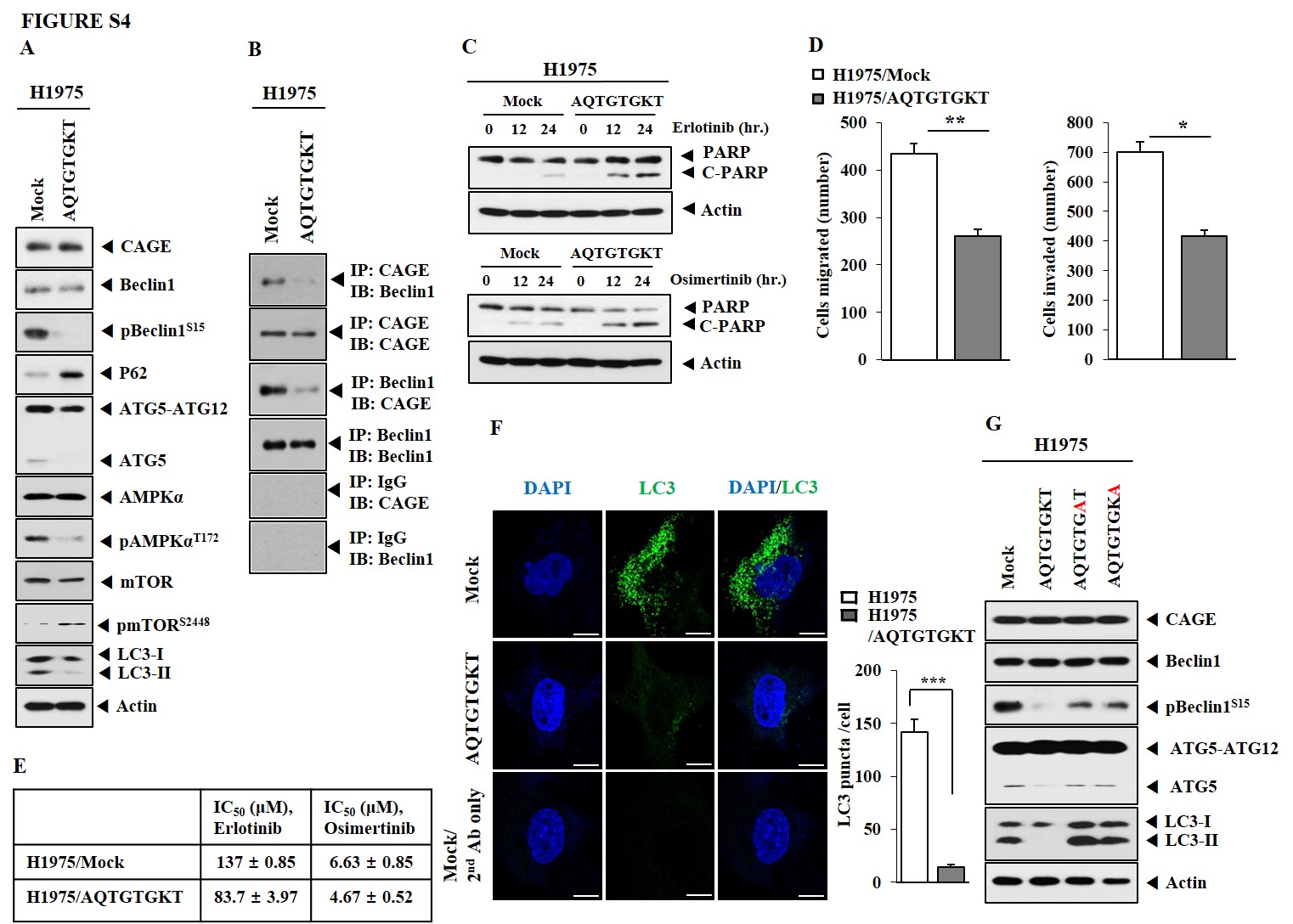

Supplement: Supplementary Figure 4 — AQTGTGKT peptide confers sensitivity to anti-cancer drugs by regulating autophagic flux in non-small cell lung cancer cells with EGFR mutation (L885R/T790 M). (A) H1975 cells were transfected with AQTGTGKT peptide (10 μM) for 24 h followed by immunoblot. (B) Same as (A) except that immunoprecipitation was performed. (C) H1975 cells were transfected with AQTGTGKT peptide (10 μM) for 24 h followed by treatment with erlotinib (20 μM) or osimertinib (5 μM) for various time intervals. Immunoblot was then performed. (D) H1975 cells were transfected with AQTGTGKT peptide (10 μM) for 48 h followed by migration and invasion assays. *p < 0.05; **p < 0.005. (E) H1975 cells were transfected with AQTGTGKT peptide (10 μM) for 24 h followed by treatment with various concentrations of erlotinib or osimertinib for 24 h. MTT assays were then performed. (F) Same as (A) except that immunofluorescence staining was performed. ***p < 0.0005. (G) H1975 cells were transfected with the indicated peptide (10 μM) for 24 h followed by immunoblot. [file Image_4.JPEG]

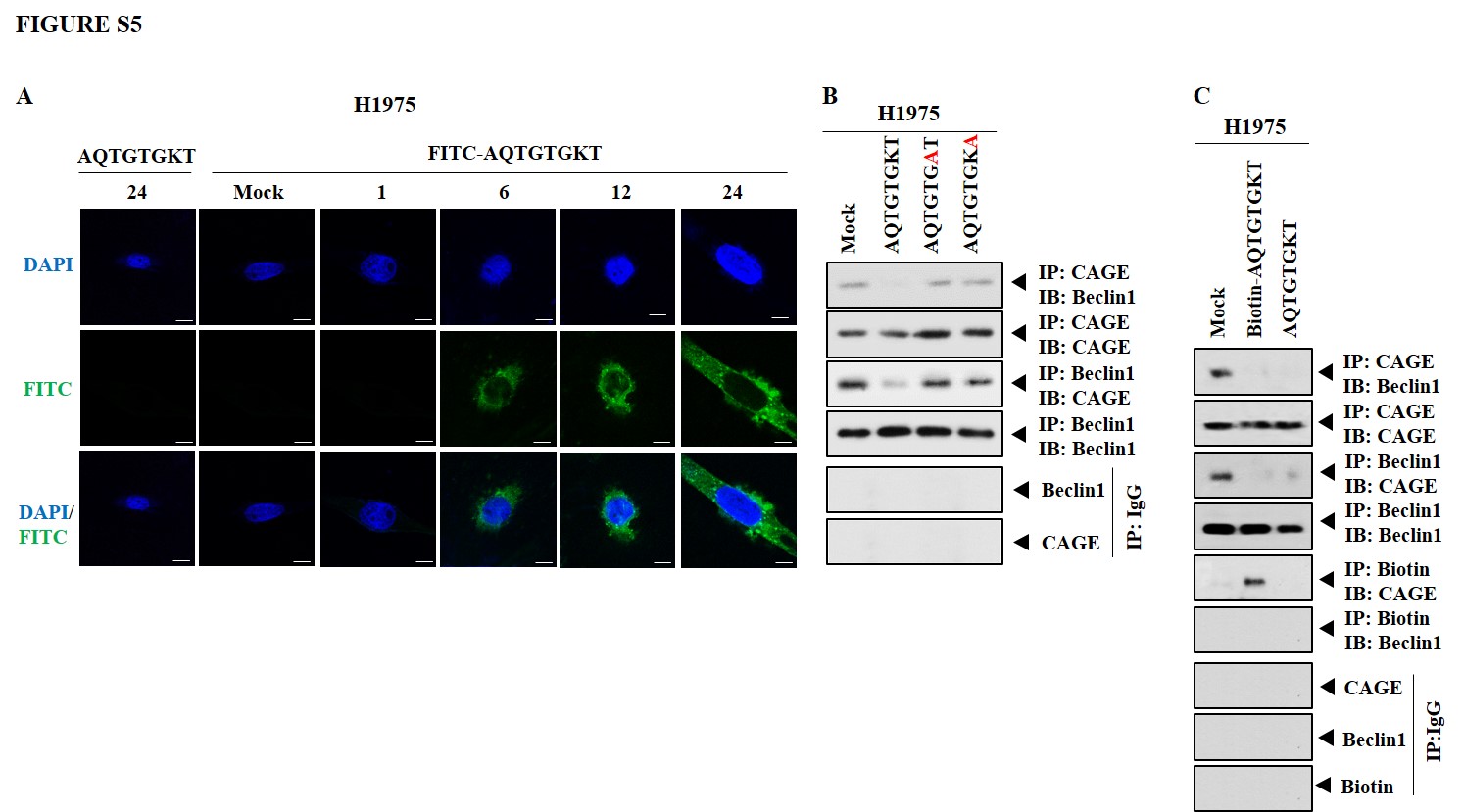

Supplement: Supplementary Figure 5 — AQTGTGKT peptide binds to CAGE and inhibits the binding of CAGE to Beclin1. (A) H1975 cells were transfected with FITC-AQTGTGKT peptide (10 μM) or unlabeled AQTGTGKT peptide (10 μM). At each time point after transfection, fluorescence microscopic observation was performed. Scale bar represents 10 μm. (B) H1975 cells were transfected with wild type or mutant CAGE-derived peptide (each at 10 μM). At 24 h after transfection, immunoprecipitation was performed. (C) H1975 cells were transfected with the indicated peptide (10 μM). At 24 h after transfection, immunoprecipitation was performed. [file Image_5.JPEG]

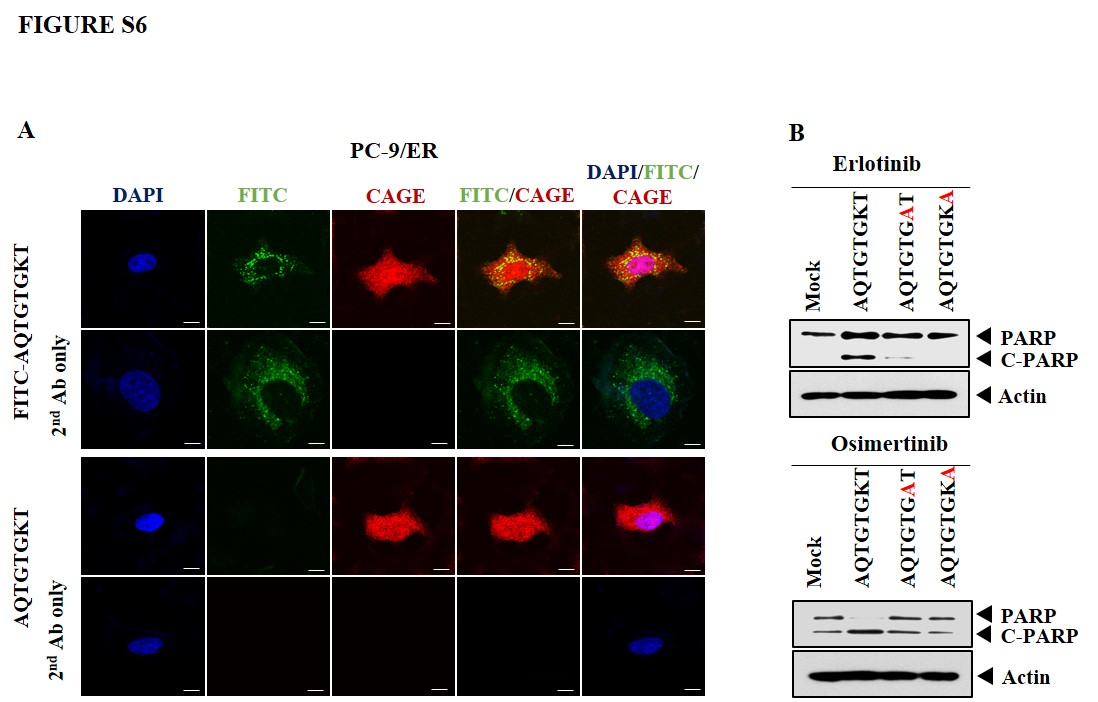

Supplement: Supplementary Figure 6 — AQTGTGKT peptide shows co-localization with CAGE and enhances cleavage of PARP in response to erlotinib and osimertinib. (A) FITC-labeled AQTGTGKT peptide (10 μM) or unlabeled AQTGTGKT peptide (10 μM) was transfected into PC-9/ER cells. At 24 h after transfection, co-localization of AQTGTGKT peptide with CAGE was examined. Scale bar represents 10 μm. (B) H1975 cells were transfected with the indicated peptide (each at 10 μM). At 24 h after transfection, cells were then treated without or with erlotinib (20 μM) or osimertiniub (5 μM) for 24 h. [file Image_6.JPEG]

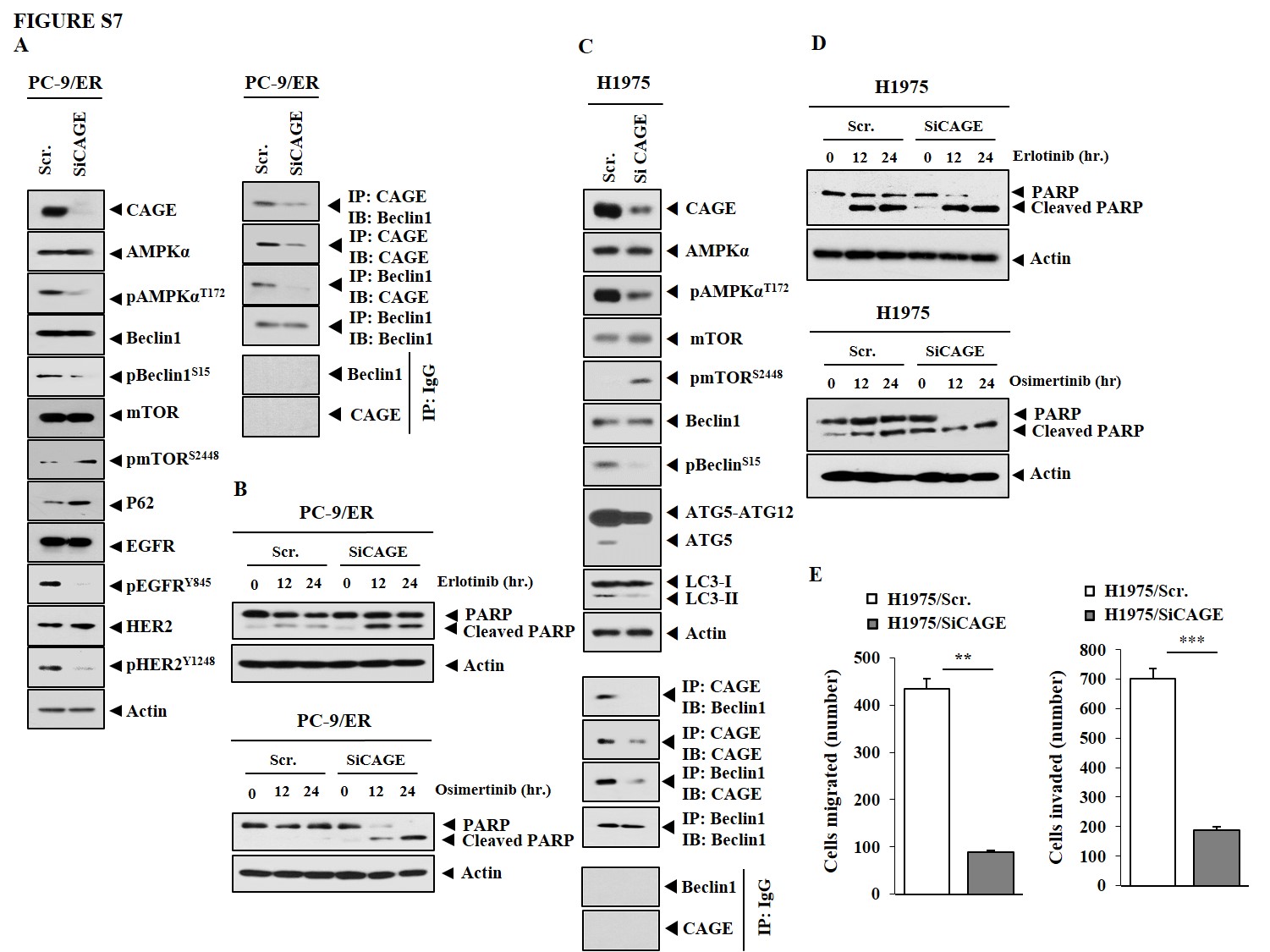

Supplement: Supplementary Figure 7 — CAGE regulates autophagic flux and anti-cancer drug-resistance. (A) PC-9/ER cell were transfected with the indicated siRNA (each at 10 nM). At 48 h after transfection, immunoblot, and immunoprecipitation were performed. (B) PC-9/ER cell were transfected with the indicated siRNA (each at 10 nM). On the next day, cells were then treated with erlotinib (20 μM) or osimertinib (5 μM) for various time intervals followed by immunoblot. (C) H1975 cells were transfected with the indicated siRNA (each at 10 nM). At 48 h after transfection, immunoblot, and immunoprecipitation were performed. (D) H1975 cells were transfected with the indicated siRNA (each at 10 nM). On the next day, cells were then treated with erlotinib (20 μM) or osimertinib (5 μM) for various time intervals followed by immunoblot. (E) H1975 cells were transfected with the indicated siRNA (each at 10 nM). At 48 h after transfection, migration, and invasion assays were performed. **p < 0.005; ***p < 0.0005. [file Image_7.JPEG]

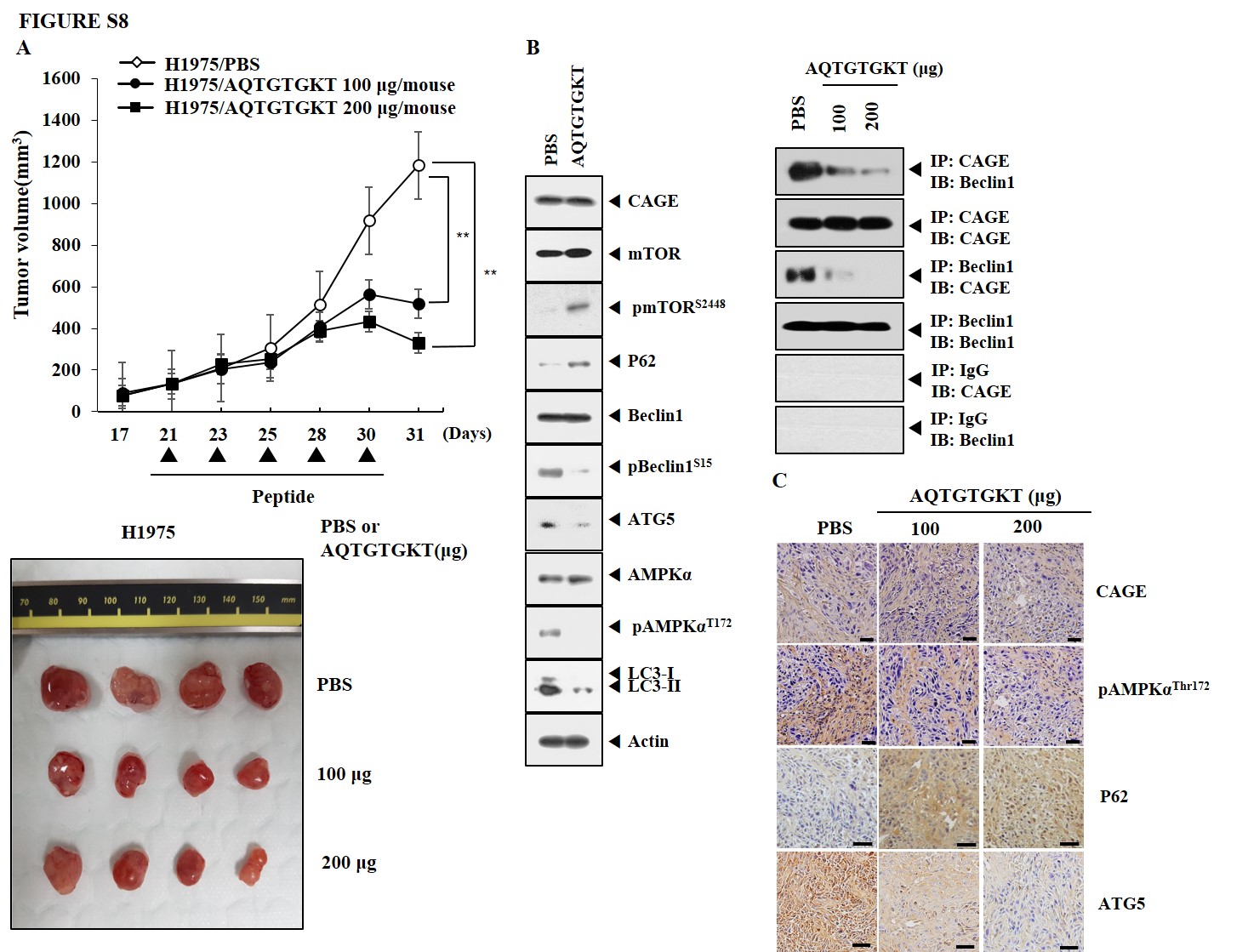

Supplement: Supplementary Figure 8 — AQTGTGKT peptide decreases tumorigenic potential of H1975 non-small cell lung cancer cells. (A) H1975 (1 × 10^6^) cells were injected into dorsal flanks of athymic nude mice. Following the establishment of sizeable tumor (~50 mm3), peptide (100, 200 μg/mouse) was injected into the tail vein five times in a total of 31 days. Each experimental group consisted of five mice. Each value represents an average obtained from the five athymic nude mice of each group. Data are expressed as a mean ± S.D. Each figure shows a representative image of mouse in each group at the time of sacrifice. **p < 0.005. (B) Lysates from the indicated tumor tissues (200 μg/mouse) were subjected to immunoblot and immunoprecipitation. (C) Immunohistochemical staining of tumor tissues employing the indicated antibody (2 μg/ml) was performed. Scale bar represents 100 μm. [file Image_8.JPEG]
